# Confrontations of the Pathogenic Fungus *Colletotrichum graminicola* With a Biocontrol Bacterium or a Ubiquitous Fungus Trigger Synthesis of Secondary Metabolites With Lead Structures of Synthetic Fungicides

**DOI:** 10.1111/1462-2920.70145

**Published:** 2025-07-14

**Authors:** Bennet Rohan Fernando Devasahayam, Henriette Uthe, Yvonne Poeschl, Holger B. Deising

**Affiliations:** ^1^ Faculty of Nutritional Sciences III, Institute of Agricultural and Nutritional Sciences, Chair of Phytopathology and Plant Protection Martin Luther University Halle‐Wittenberg Halle/Saale Germany; ^2^ EcoMetEoR, Molecular Interaction Ecology German Center for Integrative Biodiversity Research (iDiv) Halle‐Jena‐Leipzig Leipzig Germany; ^3^ MetaCom Leibniz Institute of Plant Biochemistry Halle(Saale) Germany; ^4^ German Center for Integrative Biodiversity Research (iDiv) Halle‐Jena‐Leipzig Leipzig Germany; ^5^ Biometrics and Agricultural Informatics, Faculty of Natural Sciences III Martin Luther University Halle‐Wittenberg Halle/Saale Germany

**Keywords:** *A* *spergillus nidulans*, *Bacillus amyloliquefaciens*, *Colletotrichum graminicola*, microbial biological control agents, polyketide synthases, toxic secondary metabolites

## Abstract

Microbial biological control agents are increasingly used as an alternative to synthetic pesticides. The application of these microorganisms massively affects all members of plant‐colonising microbial communities, including pathogenic fungi. In the majority of cases, the resulting competition for ecological niches is decided by the toxicity of microbial secondary metabolites (SMs) formed. In this study, we devised confrontation experiments employing the fungal maize pathogen *Colletotrichum graminicola* and antagonistic partners, that is the biocontrol bacterium *Bacillus amyloliquefaciens* and the ubiquitous ascomycete *Aspergillus nidulans*. Transcriptome studies uncovered strong de‐regulation of the vast majority of the *C. graminicola* secondary metabolite biosynthetic gene clusters (SMBGCs), with 69% and 86% of these clusters de‐regulated at confrontation sites with *B. amyloliquefaciens* or *A. nidulans* , respectively. In the biocontrol bacterium and in *A. nidulans* confronting the maize pathogen, 100% and 74% of the SMBGCs were transcriptionally de‐regulated, respectively. Correspondingly, non‐targeted high‐resolution LC–MS/MS revealed a large repertoire of 1738 and 1466 novel features formed in the fungus–bacterium and fungus–fungus confrontation, respectively. Surprisingly, several of these belong to chemical classes with lead structures of synthetic fungicides.

## Graphical abstract

Introduction of biocontrol agents to agricultural environments creates microbial confrontations. In confrontations between a fungal maize pathogen and a biocontrol bacterium or a ubiquitous fungus, novel compounds with similarity to synthetic fungicides and substances putatively toxic to humans are formed, questioning consumer safety of biological plant disease protection.

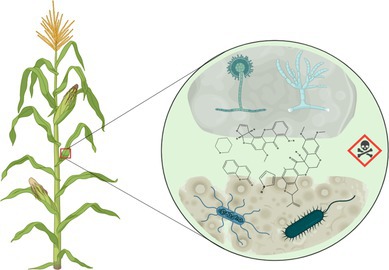

## 1 Introduction

Each year, as estimated by the Food and Agriculture Organisation (FAO), plant diseases cost the global economy approx. US$ 220 billion (http://www.fao.org/news/story/en/item/1187738/icode/), and a large body of literature highlights the role of plant pathogenic fungi in yield losses (e.g., Fisher et al. 2012; Bebber and Gurr 2015; Chaloner et al. 2021). As an example, the maize leaf anthracnose and stalk rot fungus *Colletotrichum graminicola* causes annual yield losses of approx. US$ 1 billion in the United States alone (Frey et al. 2011; O'Connell et al. 2012).

Fungicides have been used to control diseases for decades and are of paramount importance for food security in a growing world population (Beckerman et al. 2023; Brauer et al. 2019; Steinberg and Gurr 2020). Although synthetic fungicides have a leading role in food security, reports on environmental and health risks have raised concerns regarding their use in plant protection (Barber et al. 2020; Brauer et al. 2019; Crofton 1996; Jørgensen and Heick 2021; Knebel et al. 2022; Verweij et al. 2009), resulting in reduced fungicide approval rates (Brauer et al. 2019; Beckerman et al. 2023; see also https://www.pflanzenschutz‐information.de/Apps/WebObjects/PSInfoTest.woa).

As an alternative to synthetic fungicides, microorganisms exhibiting hallmark biological control activities are employed in crop disease control and have received significant global attention (Pandit et al. 2022). Importantly, the main microbial strategy of defence against confronting microorganisms is chemistry‐based, with the production of toxic secondary metabolites (SMs) impairing growth and development, or even killing the confrontation partners (Bertrand et al. 2014; Khan et al. 2020; Künzler 2018). On leaf surfaces, microbial communities can reach densities corresponding to 10^6^–10^7^ bacteria per cm^2^ and significantly lower, but nevertheless ecologically relevant numbers of fungal propagules (Leveau 2019; Lindow and Brandl 2003; Vorholt 2012). Most microorganisms are able to form a large number of SMs, the synthesis of which is mediated by secondary metabolite biosynthetic gene clusters (SMBGCs). The number of SMBGCs per genome varies dramatically across fungi, with an average of 48 clusters within the class of Eurotiomycetes, and 25% of the species within this class possess more than 60 clusters (Robey et al. 2021). SMBGC counts in bacterial genomes revealed similar numbers and variability (Wei et al. 2021). In *Bacillus* species, however, large‐scale genome mining identified an average of only 11.6 SMBGCs per genome (Yin et al. 2023). Taken together, these numbers argue that the numerous members of natural microbial communities could generate an enormous structural and functional complexity of microbial SM patterns (Collemare and Seidl 2019; Macheleidt et al. 2016; Netzker et al. 2015). Intriguingly, activation of SMBGCs and dynamics of SM production depend on community species composition and inter‐species interactions, as demonstrated in a simplified rhizosphere model including three bacterial species, that is *Bacillus cereus* , *Flavobacterium johnsoniae* and *Pseudomonas koreensis* (Chevrette et al. 2022). Also on the phylloplane, analyses of synthetic bacterial communities including typical residents of the Arabidopsis leaf surface such as *Methylobacterium* and *Sphingomonas* species suggested that higher‐order interactions strongly impact community structures (Schlechter and Remus‐Emsermann 2025), likely via SM production. Moreover, loline‐type alkaloids produced by endophytic fungal *Epichloë* species are an example of SMs positively impacting phylloplane bacterial populations. These chemicals are released onto the leaf surface, serve as major C and N resources for alkaloid‐insensitive bacteria and may drive the establishment of characteristic bacterial communities on *Epichloë*‐infected plants (Roberts and Lindow 2014). Complex and specific patterns of SMs were also found in four different *Colletotrichum* species infecting olive fruits, that is *C. acutatum* , *C. gloeosporioides*, *C. godetiae* and *C. karsti*, with clear differences observed on different olive cultivars (Riolo et al. 2023). Both SMs constitutively produced by microbes as well as SMs induced by interactions with plant‐associated microbes contribute to the diversity of chemical compounds to be expected in ecosystems. However, the species composition of agricultural ecosystems and the transcriptional responses of SMBGCs of endemic and of newly introduced biocontrol organisms are largely unknown. Yet, it can be assumed that silent SMBGCs become activated in the presence of other microorganisms, with novel SMs synthesised in several interactions (Brakhage and Schroeckh 2011; Collemare and Seidl 2019; Pidroni et al. 2018; Zhang et al. 2019). Inter‐kingdom as well as inter‐species exchanges of compounds resulting in further alterations of chemicals may generate complex patterns of novel SMs with unpredictable structure and toxicity in microbial communities to which biocontrol agents have been introduced (Deising et al. 2017; Krespach et al. 2023; Netzker et al. 2015).

Here, we established confrontations of the plant pathogenic fungus *C. graminicola* with the bacterial biocontrol agent *Bacillus amyloliquefaciens* as well as with the ascomycete fungus *Aspergillus nidulans* in order to understand mechanisms and specificities of microbial interactions. *A. nidulans* was included because of its ubiquitous occurrence and its extensively studied secondary metabolism (Caesar et al. 2020). In all confrontations, the vast majority of SMBGCs was de‐regulated, and complex transcriptional changes were reflected by an enormous repertoire of distinct newly formed chemical features, as shown by non‐targeted high‐resolution metabolome analyses. Interestingly, several compounds newly synthesised in the *C. graminicola* – *B. amyloliquefaciens* confrontation belong to the chemical classes of piperidines and cinnamaldehydes, whereas azoles were discovered in both confrontations. These compounds share their lead structures with synthetic fungicides.

## 2 Materials and Methods

### 2.1 Cultivation of Microorganisms and in Vitro Confrontation Assays

*C. graminicola* M2 (syn. M1.001) was a gift from R.L. Nicholson, Purdue University, West Lafayette, IN, USA. The maize anthracnose fungus was grown on oatmeal agar (OMA; Werner et al. 2007) or Potato Dextrose Agar (PDA; Difco, Becton, Dickinson and Company, Maryland, USA). *Bacillus amyloliquefaciens* (strain JKI‐BI‐7332/2) was provided by Ada Linkies (Julius‐Kühn‐Institute, Institute for Biological Control, Dossenheim, Germany). *A. nidulans* (strain RMS011; (Stringer et al. 1991) was provided by Vito Valiante, Hans‐Knöll‐Institute, Jena, Germany). *B. amyloliquefaciens* and *A. nidulans* were grown on PDA medium. For in vitro confrontation assays, agar discs (Ø 4 mm) from 14‐day‐old fungal or 4‐day‐old bacterial cultures were inoculated 2.5 cm apart onto PDA plates (Ø 90 mm) and grown for 12 days in darkness at 23°C. Solo‐cultures served as controls. Growth was quantified daily and plates were photographed 12 days post inoculation (dpi). To evaluate the effect of volatile inhibitors, growth assays were performed in split plates (Tuoxun Trade, Zhongshan, China, 2 compartments, Ø 90 mm) on PDA medium. All assays were conducted in triplicates.

### 2.2 Plant‐Infection Assays

*Zea mays* cv. Mikado was grown in a greenhouse for 2 weeks at 22°C± 4°C and 16 h of daylight. To confirm biocontrol activity on whole plants, leaves were sprayed with 20 mL of 5 × 10^5^ CFU/mL *B. amyloliquefaciens* (strain JKI‐BI‐7332/2) to the point of run‐off and kept at 100% relative humidity (r.h.) for 24 h. Subsequently, 20 mL of a suspension containing 10^5^ conidia of *C. graminicola* /mL were sprayed onto leaves pre‐inoculated with *B. amyloliquefaciens* and kept at 100% r.h. for 24 h. Plants only treated with bacterial or conidial suspensions served as controls. Mock‐inoculated control plants were treated with potato dextrose broth. Experiments were carried out in triplicates. Leaves were photographed at 7 dpi.

For virulence tests on leaf segments, 10 μL droplets containing 10^4^ conidia were inoculated onto intact leaf surfaces and incubated as described (Werner et al. 2007). Disease symptoms were photographed at 96 hpi. For microscopy of infection sites, leaf segments were bleached in ethanol‐acetic acid (3:1 (*v/v*)) for 24 h and examined by bright‐field microscopy (Nikon Eclipse 600, Düsseldorf, Germany).

### 2.3 Fungal Growth Inhibition Assay by IturinA and Sterigmatocystin

The inhibitory activities of Iturin A (Sigma‐Aldrich, Steinheim, Germany) and Sterigmatocystin (Sigma‐Aldrich, Steinheim, Germany) were evaluated by the Kirby‐Bauer disc diffusion assay (Bauer et al. 1959). Aliquots of 10 μL containing 50, 100, 300, 500 or 1000 μg/mL of the respective compounds were pipetted onto the filter discs. Solvents (ethanol for Iturin A or methanol for Sterigmatocystin) served as controls. Plates were incubated at 23°C for 12 days. The experiment was performed in triplicate. Inhibitory activities were evaluated as described (Skidmore and Dickinson 1976).

### 2.4 Maize Leaf Imprinting

Maize leaves obtained from the experimental station of Martin Luther University (Kühnfeld, Halle, Germany) were imprinted onto Glucose‐Yeast‐Malt (GYM) medium, as recommended by DSMZ (Braunschweig, Germany; https://bacmedia.dsmz.de/medium/65), and plates were incubated at 23°C for 2 weeks and photographed.

### 2.5 Targeted Deletion of *PKS27* of *C. graminicola*

To delete the 7481 bp of the *PKS27* gene (GLRG 10537) of *C. graminicola*, the 5′‐(981 bp) and 3′‐(979 bp) flanking regions were amplified using primer pairs PKSCgF5 and PKSCgR5, and PKSCgF3 and PKSCgR3, respectively. All primer sequences are given in Table S13. The hygromycin B phosphotransferase gene (*hph*) (Punt et al. 1987) was amplified from plasmid pAN7‐1, using primers UniHygTF and UniHygTR, and fused with the flanks by double‐joint PCR (Yu et al. 2004). The 4055 bp *PKS27* deletion cassette was amplified using primers PKSCgF5 and PKSCgR3 and transformed into *C. graminicola* protoplasts derived from oval conidia (Werner et al. 2007). Transformants were grown as described, and homologous or ectopic integration of a single copy of the deletion cassette was confirmed by Southern blotting (Werner et al. 2007). A 411 bp digoxigenin‐labelled (Roche Diagnostics, Mannheim, Germany) *PKS27*‐specific probe was amplified from genomic DNA, using primers PKSCgProbeF and PKSCgProbeR. Hybridisation and probe visualisation were done as described (Münch et al. 2011).

### 2.6 Microscopy

Differential interference contrast (DIC) and fluorescence microscopy were performed using a Nikon Eclipse 600 microscope (Nikon, Düsseldorf, Germany). Digital images were taken with a Nikon microscope camera DS‐Ri2, and image processing was performed with NIS‐Elements imaging software (Nikon, Düsseldorf, Germany).

For straining of fungal cell walls, Calcofluor White‐M2R (CFW; Sigma‐Aldrich, Steinheim, Germany) was mixed with 10% (*w/v*) KOH at a ratio of 1:1, applied to the sample, and incubated for 15 min at room temperature before inspection by fluorescence microscopy at 350 nm and 25% laser light transmission (Nikon Eclipse 600, UV‐2A filter, Düsseldorf, Germany). *Bacillus*‐induced distortions of hyphae growing on PDA at confrontation sites were examined at 12 dpi. To visualise hyphal vacuolisation, the membrane dye FM4‐64 (1 μg/mL; Thermo Fisher Scientific, Schwerte, Germany) (Vida and Emr 1995) was applied and incubated on ice in darkness for 20 min. After washing with Hank's Balanced Salt Solution (HBSS, without Ca^2+^, Mg^2+^; Thermo Scientific, Rockford, IL, USA), vacuoles were observed at 633 nm excitation wavelength and 580 to 660 nm emission wavelength (650 LP detection channel).

### 2.7 Total RNA Isolation and RNA Sequencing

PDA plates were covered with a 5.5 × 5.5 cm nylon membrane (0.45 μm, Carl Roth GmbH, Karlsruhe, Germany), inoculated as described above and incubated at 23°C for 12 days. Total RNA of *C. graminicola* or *A. nidulans* was extracted from the outer 5 mm facing the confrontation zone, using the peqGOLD Plant RNA Kit (VWR International, Leuven, Belgium). RNA isolated from the outer edges of solo‐cultures of *C. graminicola* , *A. nidulans* or *B. amyloliquefaciens* served as controls. RNA purification and concentration were performed using the GeneJET RNA cleanup and Concentration Micro Kit (Thermo Fisher Scientific, Vilnus, Lithuania).

Total RNA isolation from bacteria was carried out as described (Villa‐Rodríguez et al., 2018), with the following modifications. Bacterial cells were grown on nylon membranes (0.45 μm, Carl Roth, Karlsruhe, Germany) placed on PDA plates. From 10 plates, 5 mm segments were taken along the confrontation line and suspended in 500 μL of TE buffer (0.5 M EDTA, 1 M Tris‐Cl, pH 8.0), vortexed and centrifuged at 12,000 *x* g for 3 min at 4°C. The pellet was stored at −20°C for 1 h and homogenised by mortar and pestle. Two‐hundred μl of TE buffer containing 20 mg lysozyme/mL (Carl Roth GmbH, Karlsruhe, Germany) was added, and the suspension was vortexed and incubated at 37°C for 30 min with occasional shaking. Subsequently, 1.5 mL of TRIzol reagent (Invitrogen, Carlsbad, California, USA) was added, and the suspension was vortexed. The mixture was allowed to incubate on ice for 5 min, 300 μL chloroform (Carl Roth GmbH, Karlsruhe, Germany) was added, and the suspension was shaken vigorously. After centrifugation at 13,000 *x* g for 15 min at −3°C, the aqueous phase was mixed with the two‐fold volume of isopropanol (Carl Roth GmbH, Karlsruhe, Germany), and RNA was precipitated at −80°C overnight. The precipitate was centrifuged at 13,000 *x* g for 15 min at −3°C. The resulting pellet was washed thrice with 1 mL of ice‐cold 75% (*v/v*) ethanol (Carl Roth GmbH, Karlsruhe, Germany) and centrifuged at 13,000 *x* g for 5 min at −3°C. The RNA pellet was air‐dried and dissolved in nuclease‐free water (Ambion, Life Technologies, Carlsbad, CA, USA).

RNA quality control, mRNA library construction and sequencing were performed by Genewiz (Azenta Life Sciences, Leipzig, Germany).

### 2.8 Quality Control, Mapping and Quantification of Reads

Identification of differentially expressed genes (DEGs) and all other applications were used as described (https://usegalaxy.eu/) (Batut et al. 2018). The quality of raw reads was analysed using FastQC v0.73. Adapters and low‐quality reads were trimmed by the Trimmomatic tool v0.38.1. Clean reads were mapped to the *C. graminicola* _M1_001_V1 reference genome (https://fungi.ensembl.org/Colletotrichum_graminicola/Info/Index ; accessed on July 20, 2022), using RNA STAR v2.7.8a. *Aspergillus nidulans* reads were mapped to the *A. nidulans* FGSC A4 reference genome (NCBI; accessed on 14 September 2022; https://www.ncbi.nlm.nih.gov/genome/17?genome_assembly_id=299190). Bacterial clean reads were mapped to the *B. amyloliquefaciens* DSM 7 reference genome (NCBI; https://www.ncbi.nlm.nih.gov/datasets/genome/GCF_000196735.1/; accessed on 12 February 2024). Reads per gene were counted using featureCounts v2.0.1.

### 2.9 Identification and Classification of Differentially Expressed Genes (DEGs)

DEGs were obtained using DESeq2 v2.11.40.7 with a cutoff of an adjusted *p*‐value < 0.05, fold change (FC) > 2 for increased and FC < 0.5 for decreased transcript abundances. Principal component analysis (PCA) plots and correlation matrices were also prepared using the DESeq2 package. Venn diagrams of DEGs were generated using BioVenn (https://www.biovenn.nl/) (Hulsen et al. 2008).

### 2.10 Identification of Secondary Metabolite Biosynthesis Gene Clusters (SMBGCs)

Function and location of genes in SMBGCs of *C. graminicola* were defined using NCBI and Cytoscape String (Doncheva et al. 2018). Genes encoding polyketide synthases (PKS), non‐ribosomal peptide synthetases (NRPS) and genes harboured by indole or terpene biosynthesis clusters were identified based on the reference genome of *C. graminicola* (O'Connell et al. 2012). For *A. nidulans* , the FGSC A4 reference genome was used to map differentially expressed SM genes to their corresponding SMBGCs (Inglis et al. 2013; Soukup et al. 2012). In the case of *B. amyloliquefaciens* , the reference genome of strain DSM7 (https://www.ncbi.nlm.nih.gov/datasets/genome/GCF_000196735.1/) was uploaded into antiSMASH (https://antismash.secondarymetabolites.org/#!/start; Blin et al. 2023) to identify its SMBGCs. Differentially expressed SM genes obtained from transcriptome analysis were subsequently mapped to the identified SMBGCs.

### 2.11 Gene Ontology Enrichment Analyses

To functionally characterise the DEGs, Gene Ontology (GO) enrichment analyses were performed with a focus on the Molecular Function (GO:MF) category. For *C. graminicola* and *A. nidulans* , enrichment analyses were conducted using gProfiler (https://b2t.cs.ut.ee/gprofiler/gost) with default parameters, by uploading the respective gene IDs into the server interface (Raudvere et al. 2019). In the case of *Bacillus amyloliquefaciens* , the DEG lists were analysed using the ShinyGO v0.80 tool (http://bioinformatics.sdstate.edu/go/) (Ge et al. 2019) to retrieve GO annotations. The enriched GO:MF terms were visualised as a bubble plot using R (version 4.3.1) (R Core Team, 2022), with the aid of the packages ggplot2 (H. Wickham 2016), dplyr and forcats (H. Wickham 2023).

### 2.12 Quantification of Transcript Abundances by RT‐qPCR

RT‐qPCR primers (Table S13) were designed based on the corresponding gene sequences (https://www.ncbi.nlm.nih.gov/), using Clone Manager 9 (Sci‐Ed, Cary, NC, USA). The lengths of primers and amplicons were 20 bp and 150–200 bp, respectively. Single melting curve peaks confirmed the specificity of all primer pairs. RT‐qPCRs were carried out using the iTaq Universal SYBR Green One‐Step kit (Bio‐Rad Laboratories, Feldkirchen, Germany). Transcripts of *act1* and *gyrA* were used as reference genes for fungi (Gao et al. 2011; Krijger et al. 2008) and *B. amyloliquefaciens* (Liu et al. 2022), respectively. Each reaction was performed using three independent biological and three technical replicates. The relative fold change of transcript abundances was calculated as 2^−ΔΔCt^ values (Livak and Schmittgen 2001).

### 2.13 Secondary Metabolite Extraction

The extraction of SMs from PDA plates was done as described (Nickles et al. 2021), with modifications. In brief, at 12 dpi, 5 mm stripes were taken from the colony edges at confrontation sites as well as from the area of the confrontation zone that had not been colonised by microbes (100 Petri dishes) and transferred into Erlenmeyer flasks. Samples from solo‐cultures (10 Petri dishes) served as control. Samples were homogenised in ethyl acetate, using an Ultra‐Turrax homogeniser (T8.01, IKA Labortechnik, Staufen, Germany); suspensions were kept in a shaker (Unitron, Infors AG, Bottmingen, Switzerland) at RT for 3 h at 200 rpm and were then filtered (Whatman cellulose round filters, Ø 125 mm, Carl Roth, Karlsruhe, Germany). Extracts were dried in a rotary evaporator at 45°C and 240 mbar (Laborota 4000, Heidolph Instruments GmbH, Schwabach, Germany). The resultant crude extracts were transferred to a glass vial, and samples were adjusted to a concentration of 1 mg/ml methanol.

### 2.14 LC–MS/MS Analysis

LC‐ESI‐Q‐ToF‐MS measurements were based on methods described by (Böttcher et al. 2009). Chromatographic separations were performed at 40°C on an UltiMate 3000 Standard Ultra‐High‐Pressure Liquid Chromatography system (Thermo Fisher Scientific, Vilnus, Lithuania) equipped with an Acclaim Rapid Separation Liquid Chromatography (RSLC) 120 column (150 x 2.1 mm, particle size 2.2 μm, Thermo Fisher Scientific, Vilnus, Lithuania). The following gradient was used at a flow rate of 0.4 mL/min: 0–1 min, isocratic 95% A [water/formic acid 99.95/0.05 (*v/v* %)], 5% B [acetonitrile/formic acid 99.95/0.05 (*v/v* %)]; 1–2 min, linear gradient from 5% to 20% B; 3–8 min, linear gradient from 20% to 25% B; 8–16 min, linear gradient from 25% to 95% B; 16–18 min, isocratic 95% B; 18–18.01 min, linear from 95% to 5% B; and 18.01–20 min, isocratic 5% B. The injection volume was 3 μL (full loop injection). Eluted features were detected from m/z 90 to 1600 at a spectra rate of 5 Hz using an ESI‐UHR‐Q‐ToF‐MS (maXis impact, Bruker Daltonics, Bremen, Germany) equipped with an Apollo II electrospray ion source in positive ion mode. Calibration of the m/z scale was performed for individual raw data files on sodium formate cluster ions obtained by automatic infusion of 1.66 μL/min of 10 mM sodium formate solution of NaOH in 50/50 (*v/v* %) isopropanol/water containing 0.2% (*v/v*) formic acid at the end of the gradient (HPC mode).

LC–MS and MS/MS data were processed with MetaboScape 4.0 (https://www.bruker.com/en/products‐and‐solutions/mass‐spectrometry/ms‐software.html), using Bruker's T‐ReX 3D algorithm with the following settings: intensity threshold 1500 counts, minimum peak length 7 spectra, feature signal = intensity, and mass recalibration auto‐detect. Recursive feature extraction: minimum peak length (recursive) 5 spectra, a minimum number of features for recursive extraction 1 of 8. Bucket filter: presence of features in a minimum number of analyses 1 of 8.

Annotation of features was based on 1) an in‐house library of analytical standards and known metabolites according to mass, retention time and spectrum, 2) the KNApSAcK family (Afendi et al. 2012) considering mass and spectral similarity to compound class and 3) via spectral similarity to the databases NIST17, WEIZMASS (Shahaf et al. 2016), Sumner Spectral library (https://www.bruker.com/de/products‐and‐solutions/mass‐spectrometry/ms‐software/metabolomics‐spectral‐libraries.html), MoNA (https://mona.fiehnlab.ucdavis.edu/), GNPS (https://gnps.ucsd.edu/), ReSpect (Sawada et al. 2012) and an in‐house database via the spectral library search function of MetaboScape.

To predict compound classes, the software framework Sirius (v5.8.6.) with the integrated tools CSI:FingerID, CANOPUS and ZODIAC was used, and De Novo Sum formula prediction with Sirius was used with the following parameters:

Possible ionisations [M + H]+, [M + K]+, [M + Na]+, filter for isotope patterns, ignore MS/MS isotope scorer, MS2 Mass Dev 5 ppm, 10 candidates, 1 candidate per ion, 60s tree timeout, 0 compound timeout, use heuristic above 300 m/z, use heuristic only above 650 m/z; allowed elements in Molecular Formula, S (5), O (15), N (15), P (5), C and H infinite, Cl, and B automatic.

For molecular formula ranking, the following ZODIAC parameters were used: considered candidates 300 m/z = 10, considered candidates 800 m/z = 50, using 2‐step approach, edge threshold 0.95, minimum local connections = 10, Gibbs sampling iterations = 20.000, burn‐in = 2.000 and separate runs = 10. Parameters for the structure elucidation using CSI:FingerID included fallback adducts [M + H]^+^, [M‐H2O + H]^+^, [M + K]^+^ and [M + Na]^+^. The databases used were BioDatabases, BioCyc, METACYC, CHEBI, COCONUT, ECOCYCMINE, GNPS, KEGG, KEGGMINE, KNAPSACK, MACONDA, MESH, UNDP, PLANTCYC, PUBCHEM, YMDB, YMDB Mine, ZINCBIO and ADDITIONAL.

### 2.15 Post‐Processing of LC–MS Data

A feature table was post‐processed using the R program (RCoreTeam 2022; https://www.R‐project.org/) by removing features detected in acetonitrile (ACN; blank) and PDA (medium) samples. To account for unreliable measurements, uncertain intensities below 1500 were set to 0. The threshold of 1500 corresponds to the same value used in MetaboScape. A resulting feature table was used to determine confrontation‐specific features. Features also occurring in the solo samples of the confrontation partners were removed (Figure S9) to retain only confrontation‐specific features.

To create one composite feature table out of two assay‐specific feature tables, tables were first sorted by retention time (rt). Afterwards, corresponding features uniquely identified by pairs of mass‐to‐charge ratio (mz) and retention time (rt) values were determined in both tables by allowing a deviation of 0.001 in mz values and a shift of at most 10 s in retention time.

The composite feature table was used for comparative analyses and visualisation with alluvial plots.

Features are uniquely characterised by pairs of mz and rt values and can be regarded as putative compounds. Features annotated by their chemical class are referred to as compounds. Also, features annotated with the current state of the annotation databases are referred to as (putative) compounds.

### 2.16 Statistical Analyses and Bioinformatics

Statistical differences between groups were analysed using a single‐factor ANOVA test, followed by Tukey‐HSD test, with an alpha degree of *p* < 0.05. Data were assessed with Excel 2016 and R (RCoreTeam 2022; https://www.R‐project.org/), along with packages: ggplot2 (H. Wickham 2016) for generating violin plots, line plots and bar plots. Plotly (Sievert 2020) was used for generating sunburst plots, ggalluvial (https://corybrunson.github.io/ggalluvial/articles/ggalluvial.html) (Brunson 2017) was used for constructing alluvial plots, and eulerr (https://cran.rproject.org/web/packages/eulerr/index.html) (Larsson and Gustafsson 2018) for generating Venn diagrams.

Based on their intensities across the samples, features were assigned to specific pre‐defined profiles by applying the Profile‐Interaction‐Finder (Poeschl et al. 2014; Trenner et al. 2017).

## 3 Results

### 3.1 Varying Phenotypes Occur in Confrontations Between the Maize Pathogen *C. graminicola* and the Biocontrol Bacterium *B. amyloliquefaciens* or the Saprophytic Fungus *A. nidulans*

**FIGURE 1:**
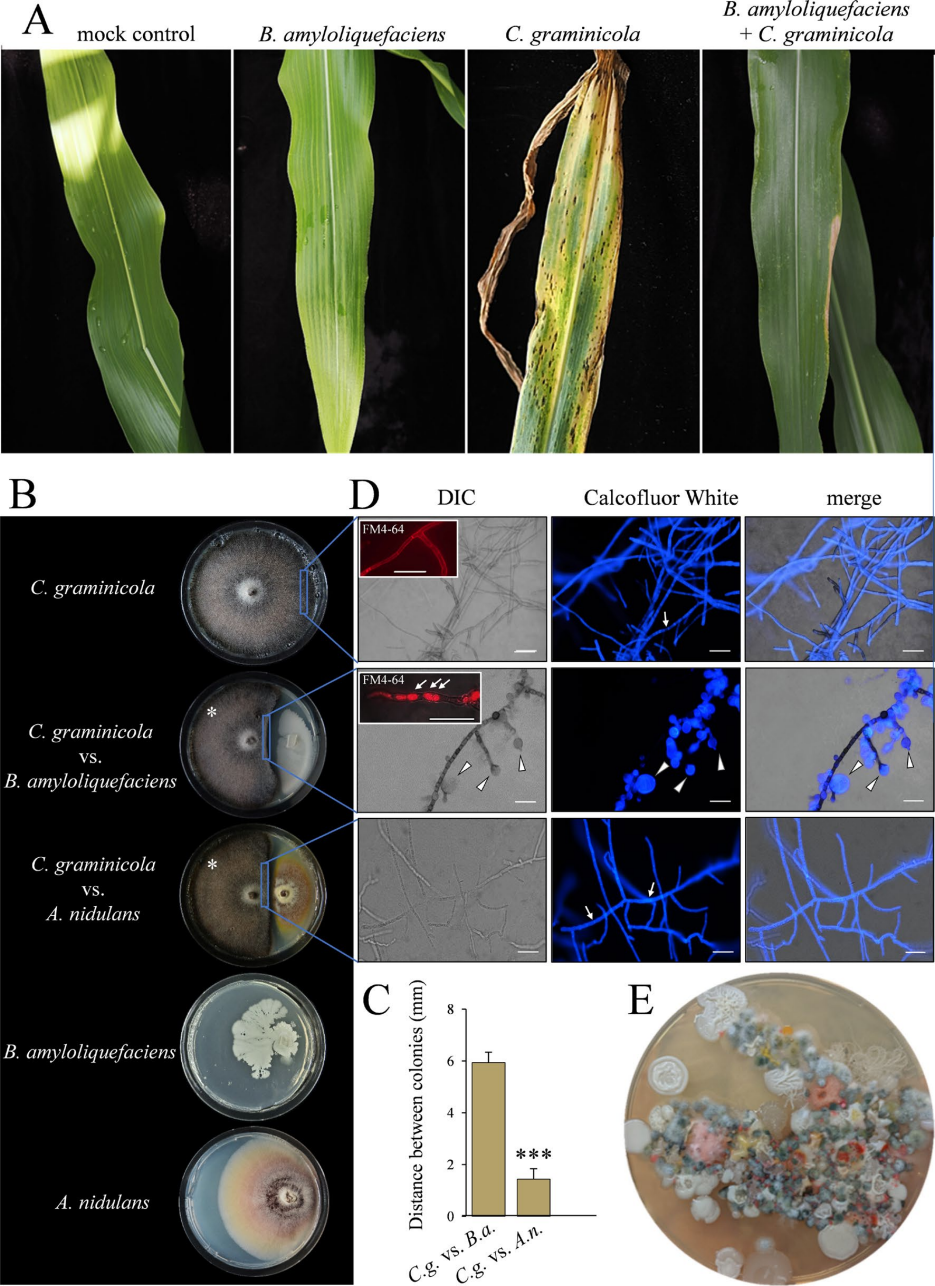
In vitro confrontation assay with the maize pathogen *C. graminicola* versus the biocontrol bacterium *B. amyloliquefaciens* or the fungus *A. nidulans* . (A) Maize leaves inoculated with *C. graminicola* showed severe anthracnose disease symptoms, but leaves pretreated with *B. amyloliquefaciens* did not. Leaves only treated with *B. amyloliquefaciens* and mock‐inoculated leaves showed no disease symptoms. Photos were taken at 7 dpi. (B) Confrontation assay between *C. graminicola* and *B. amyloliquefaciens* or *A. nidulans* on PDA indicate distance inhibition. White asterisks indicate *C. graminicola* . Photos were taken at 12 dpi. (C) Separation between microbial colonies reveals growth inhibition at 12 dpi. (D) Microscopy shows that hyphae of *C. graminicola* form swellings in confrontation with *B. amyloliquefaciens* , but not in confrontation with *A. nidulans*, or in solo‐cultures. The cell wall dye Calcofluor White allows visualisation of hyphal swellings in the confrontation between *C. graminicola* and *B. amyloliquefaciens* (Calcofluor White; arrowheads) and thin hyphae with regularly spaced septae (Calcofluor White; small arrows). The membrane dye FM4‐64 (DIC, inserts) highlights vacuolisation in the confrontation with *B. amyloliquefaciens* (FM4‐64, arrows). Photos were taken at 12 dpi. The merged image displays the overlay of DIC and CFW channels. Scale bar represents 50 μm. (E) A maize leaf imprint on GYM medium showing diverse bacterial colonies contacting each other without inhibition zones. Experiments were performed in triplicate. Error bars represent + SD.

The biocontrol bacterium *B. amyloliquefaciens* effectively inhibits anthracnose disease symptom expression caused by the pathogen *C. graminicola* . While the pathogen causes massive anthracnose disease symptoms, the fungus is unable to cause disease on leaves pre‐inoculated with *B. amyloliquefaciens* (Figure 1A; compare *C. graminicola* and *B. amyloliquefaciens* *+* *C. graminicola* ). On mock‐inoculated leaves and on leaves inoculated with *B. amyloliquefaciens* alone, disease symptoms were not observed (Figure 1A; mock control and *B. amyloliquefaciens* ). To characterise microbial interactions, confrontations between the maize anthracnose fungus *C. graminicola* and *B. amyloliquefaciens* or the non‐pathogenic model fungus *A. nidulans* were established in vitro (Figure 1B). In both interactions, a non‐colonised area occurred between confrontation partners, referred to as distance inhibition (Bertrand et al. 2014), suggesting secretion of inhibitory molecules (Figure 1B,C). The fact that growth inhibition occurred neither at the side opposite of the confrontation zone, nor on split Petri dishes, argues that the inhibitory molecules are not volatile (Figures 1B and S1). Differential interference contrast (DIC) and fluorescence microscopy of Calcofluor White‐ or FM4‐64‐stained hyphae of *C. graminicola* confronting *B. amyloliquefaciens* revealed massive hyphal swellings (Figure 1D, arrowheads) and vacuolation (Figure 1D, arrows in insert). Both hyphal swellings and vacuolation are known to be induced in fungal hyphae by bacterial cyclic lipopeptides such as iturin A and/or plipastatin A, respectively (Gong et al. 2015). Indeed, large swellings were also observed in hyphae of *C. graminicola* growing in the vicinity of filter disks soaked with commercially available iturin A (Figure S2A; Iturin A; S2B). Quantifying hyphal protrusions at increasing distances from the border of the *C. graminicola* colony revealed that cell wall‐challenging lipopeptides and likely other molecules had migrated up to ~1 cm into the colonies of confrontation partners at 12 days post inoculation (dpi) (Figure S3). By contrast, hyphae of *C. graminicola* from control plates (Figure 1D; *C. graminicola* ) and from confrontations with *A. nidulans* (Figure 1D; *C. graminicola* vs. *A. nidulans* ) showed normal septate filaments (Figure 1D; Calcofluor White, arrows), suggesting that in the fungus–fungus confrontation cell wall biogenesis is not a prime target. The fact that growth inhibition occurred further suggests that secretion of toxic SMs not affecting hyphal morphology caused growth inhibition. Imprinting environmental communities present on maize leaf surfaces onto a Petri dish resulted in colonies that expanded until they contacted each other and only rarely showed inhibition zones (Figure 1E). The fact that inhibition zones are not or only rarely present in the interactions isolated from leaf surfaces is important to note because it suggests that in most interactions toxic SMs are not secreted.

Collectively, these data show that the growth of confrontation partners of the *C. graminicola* – *B. amyloliquefaciens* and of the *C. graminicola* – *A. nidulans* interaction is strongly inhibited at the confrontation zone, resulting in distance inhibition. Moreover, these data suggest that non‐volatile, secreted inhibitory compounds determine the outcome of the interactions of the maize anthracnose fungus with either a biocontrol bacterium or a ubiquitous fungus.

### 3.2 The Confrontational Transcriptome Highlights Interaction‐Specific SMBGC Responses

To identify genes de‐regulated in confrontations of *C. graminicola* with *B. amyloliquefaciens* or *A. nidulans* , mRNA was isolated from the peripheral 5 mm of colonies of three independent repeats of each partner facing the confrontation zone. RNA extracted from margins of three independent solo‐cultures of *C. graminicola* , *B. amyloliquefaciens* and *A. nidulans* growing in the absence of a confrontation partner served as controls. The three independent libraries of *C. graminicola, B. amyloliquefaciens* and *A. nidulans* solo‐cultures yielded a total of 23.9, 32.2 and 32.7 million clean reads, respectively. From *C. graminicola* confronting *B. amyloliquefaciens* or *A. nidulans* , 43.2 and 33.5 million clean reads were obtained, and 40.2 and 34.6 million clean reads were obtained for *B. amyloliquefaciens* and *A. nidulans* , respectively. Clean reads were mapped to the 12,399 genes of the *C. graminicola* reference genome, and to the 4147 and 10,518 genes of the *B. amyloliquefaciens* and *A. nidulans* reference genomes, respectively. Differentially expressed genes (DEGs) in individual microbes in distinct confrontations were identified by DESeq2, with a fold change (FC) of > 2 defined as up‐regulated, and a FC of < 0.5 as down‐regulated (Table S1–S4). The term de‐regulated genes is collectively used to include both up‐ and down‐regulated genes.

Principal component analyses (PCA) confirmed clear separation of three independent biological repeats of pure cultures of *C. graminicola* (*Cg*), *B. amyloliquefaciens* (*Ba*) and *A. nidulans* (*An*) and the *C. graminicola* – *B. amyloliquefaciens* (*Cg* vs. *Ba*) or *C. graminicola* – *A. nidulans* (*Cg* vs. *An*) confrontations (Figure S4A–S4C). The dissimilarities between the groups were supported by heatmaps of the correlation matrices, with sample clustering based on normalised transcript counts (Figure S4D–S4F). In all confrontations, more genes showed decreased than increased transcript abundances for all partners. In *C. graminicola* confrontations with *B. amyloliquefaciens* or *A. nidulans*, a total of 1606 and 2389 DEGs were identified. In the maize pathogen, 570 DEGs showed increased and 1036 showed decreased transcript abundances, respectively, in the confrontation with the bacterium, and 892 DEGs showed increased and 1497 decreased transcript abundance, respectively, in the confrontation with *A. nidulans* (Figure S4G and Tables S1 and S3). A total of 935 DEGs were specific for the *C. graminicola* – *B. amyloliquefaciens* confrontation, and 1718 distinct DEGs were identified in the *C. graminicola* – *A. nidulans* confrontation. The specificity of the responses in the fungus–bacterium and fungus–fungus interactions was emphasised by only 671 DEGs shared in both confrontations (Figure S4H and Table S5). Intriguingly, 82 of all the DEGs of *Colletotrichum* in the confrontation with *B. amyloliquefaciens* and 116 of those in the confrontation with *A. nidulans* belonged to the category of secondary metabolism (SM) genes. Thus, more than 50% of the 300 *C. graminicola* genes categorised as SM genes (O'Connell et al. 2012) were transcriptionally de‐regulated in the microbial confrontations studied, with only 12% of the SMBGC genes de‐regulated in both confrontations (Figure S4I). While more SM genes showed increased transcript abundances in the confrontation partners, more SM genes showed decreased transcript concentrations in *C. graminicola* (Figure S4J).

Due to the establishment of distance inhibition in the confrontations of *C. graminicola* with the bacterial and with the fungal partner (Figure 1B,C), and due to the prominent response of some SM genes (Figure S4I and Table S6), we decided to study the entire repertoire of genes organised in SMBGCs in more detail.

**FIGURE 2:**
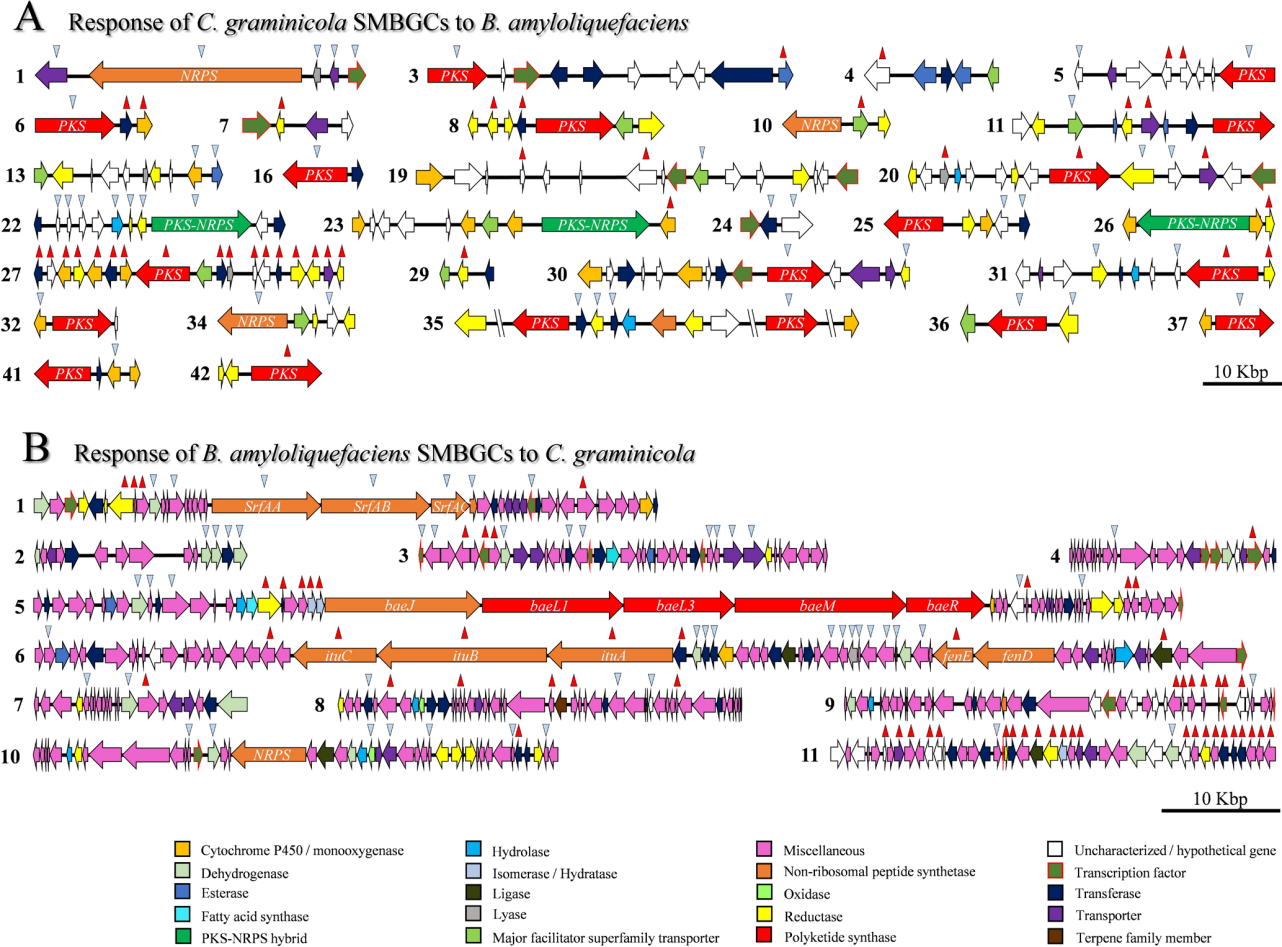
Physical map of de‐regulated SMBGCs in the *C. graminicola–B. amyloliquefaciens* confrontation. Horizontal arrows represent SM genes and their transcriptional direction. The putative function is indicated by the colour code. Red and blue arrowheads denote increased or decreased transcript abundances in the confrontation. Scale bar represents 10 kb. (A) In *C. graminicola* , 29 out of 42 SMBGCs harbour de‐regulated genes in confrontation with *B. amyloliquefaciens* . (B) During the confrontation between *B. amyloliquefaciens* and *C. graminicola* , all 11 SMBGCs of *B. amyloliquefaciens* harbour de‐regulated genes.

**FIGURE 3:**
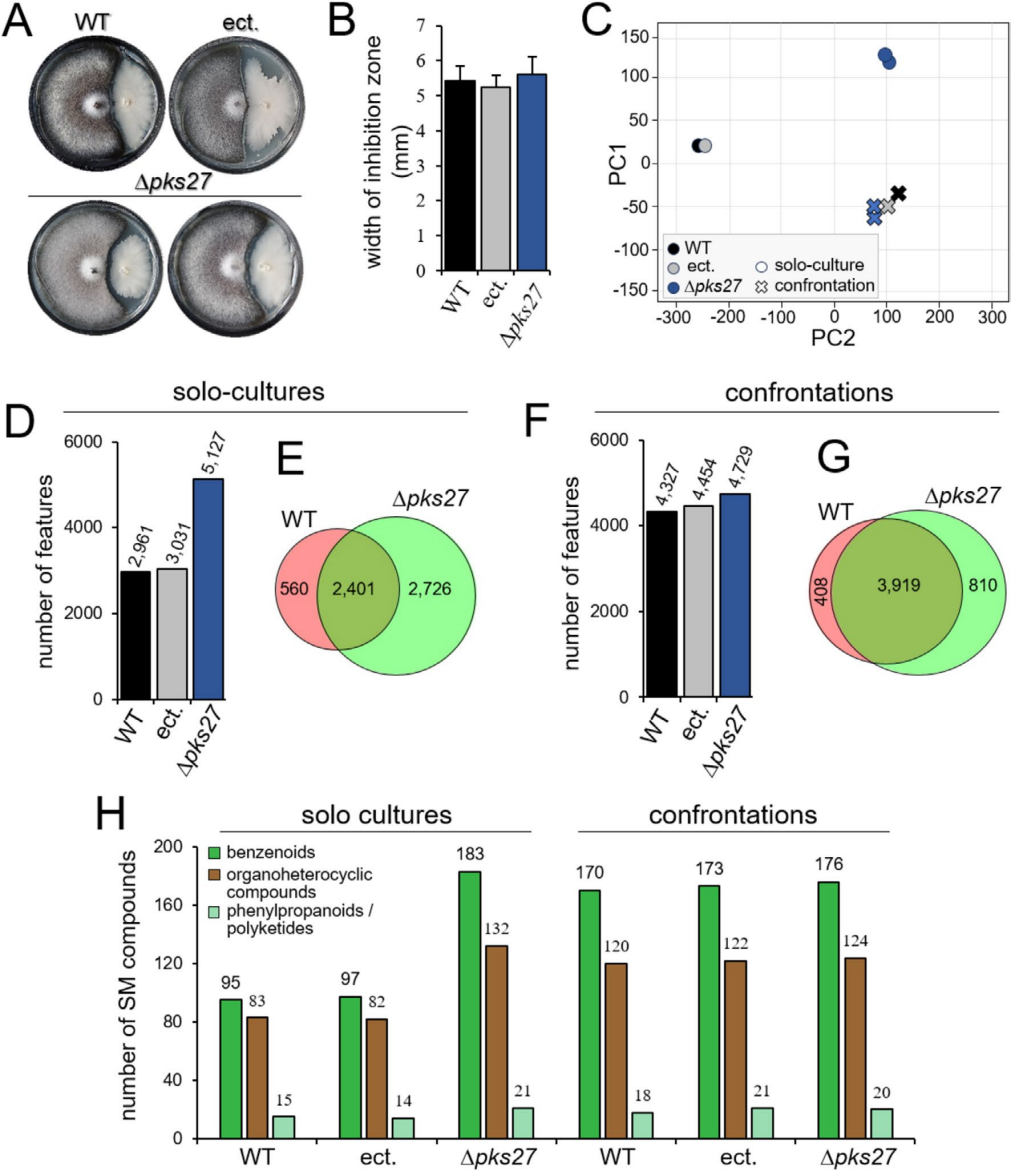
Comparative analysis of SM production in WT and ∆*pks27* mutants of *C. graminicola* under solo‐culture and confrontations conditions. (A) In vitro confrontation assays between WT, ectopic (ect.) and ∆*pks27* strains on PDA medium. Photos were taken at 12 dpi. (B) Quantification of inhibition zones indicates that *PKS27* does not affect confrontations of *C. graminicola* WT, ectopic (ect.) and ∆*pks27* strains with *B. amyloliquefaciens* . Error bars are +SDs. (C) PCA plot based on extracted metabolites show the separation of WT, ectopic (ect.) and ∆*pks27* strains growing in solo‐cultures, while close clustering of all strains occurred in confrontations. (D) Quantification of feature numbers under solo conditions, with the ∆*pks27* strains displaying significantly more features than the WT and ectopic (ect.) strains. (E) Venn diagram illustrating unique and shared features between WT and ∆*pks27* strains under solo‐growth conditions. (F) Quantification of feature numbers in confrontations, showing similar feature numbers produced by both WT, ectopic (ect.) and ∆*pks27* strains. (G) Venn diagram showing a strong increase in features shared between *C. graminicola* WT and ∆*pks27* strains during confrontation with *B. amyloliquefaciens* . (H) Quantification of the number of benzenoids, organoheterocyclic compounds, phenylpropanoids and polyketides produced by WT, ectopic (ect.) and ∆*pks27* strains under solo‐growth conditions. Under confrontation, all genotypes showed similar numbers of SMs produced.

**FIGURE 4:**
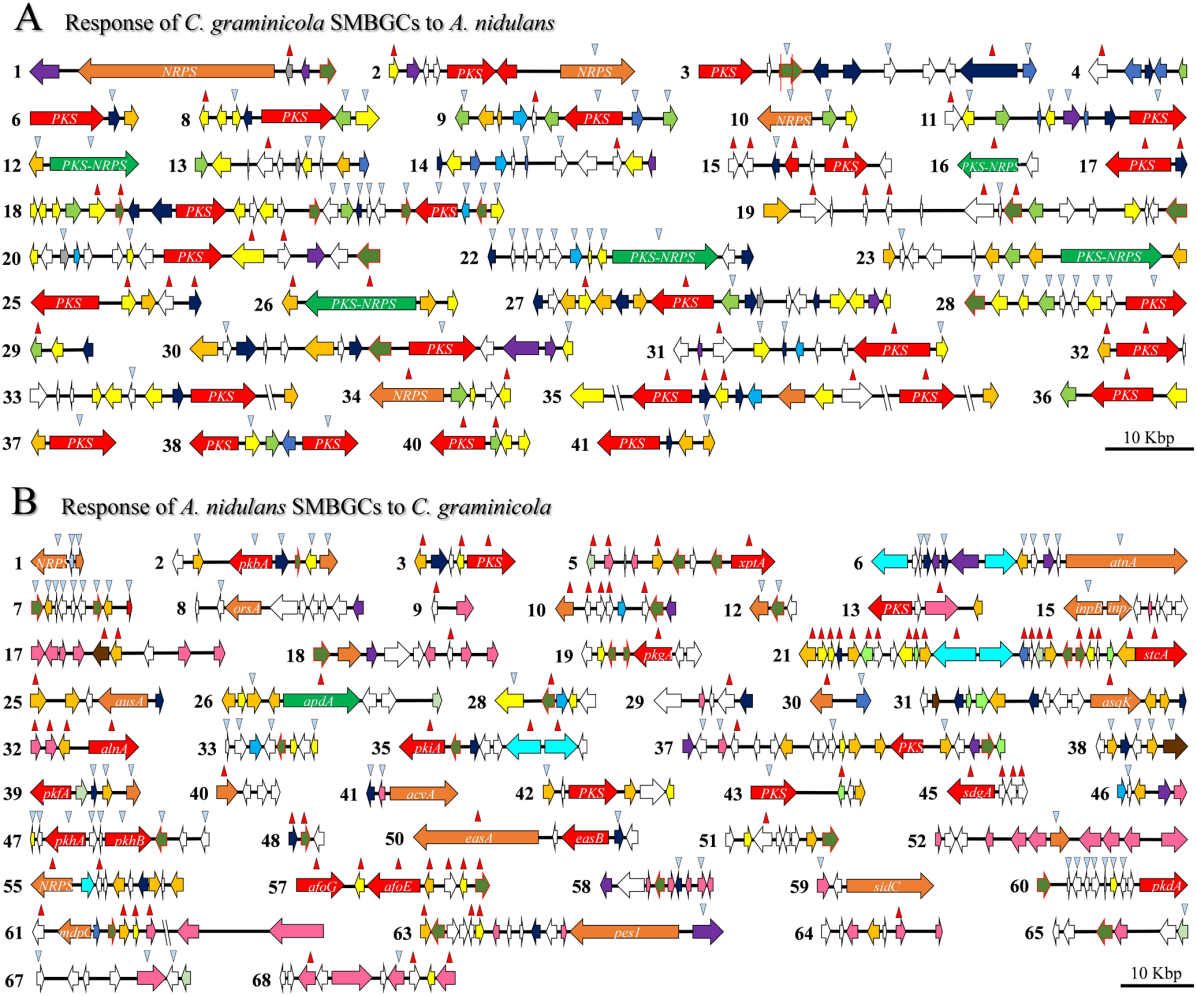
Physical map of de‐regulated SMBGCs in the *C. graminicola* – *A. nidulans* confrontation. The horizontal arrows represent SM genes and their transcriptional direction. Putative function as indicated by the colour code is as in Figure 2. Red and blue arrowheads denote increased or decreased transcript abundances in the confrontation. The scale bar represents 10 kb. (A) In *C. graminicola* , 36 out of 42 SM SMBGCs harbour de‐regulated genes in confrontation with *A. nidulans* . (B) In *A. nidulans* confronting *C. graminicola* , 50 out of the total of 68 SM clusters are transcriptionally de‐regulated.

In *C. graminicola* , 300 genes belong to 42 SMBGCs (O'Connell et al. 2012). Twenty‐nine and 36 of these harbour at least one transcriptionally de‐regulated gene in the confrontation with *B. amyloliquefaciens* or *A. nidulans* , respectively (Figures 2A and 4A, and Table S7). In the confrontation with *B. amyloliquefaciens* , 40 SM genes present in 29 SMBGCs of *C. graminicola* showed increased and 42 showed reduced transcript abundances (Figures 2A and S4J). The de‐regulated genes included 12 polyketide synthase (PKS) genes, 2 non‐ribosomal peptide synthetase (NRPS) genes and 1 PKS‐NRPS hybrid gene. Interestingly, in SMBGC 27, harbouring a core PKS gene designated as *PKS27* (GLRG 10537), 17 out of 18 SM genes showed increased transcript concentrations (Figure 2A and S5A). As SMBGC 27 was the only widely up‐regulated SMBGC, we hypothesised that its product(s) may contribute to the establishment of distance inhibition. Therefore, in order to test this hypothesis, we deleted the core *PKS* gene of this cluster (Figure S6). Two independent Δ*pks27* mutants tested did not exhibit any defects in colony phenotype, growth rates, conidiation, appressorium differentiation and virulence on excised maize leaf segments (Figure S7). Surprisingly, and in contrast to the above hypothesis, the distance of the confrontation zones established between the WT, ectopic and Δ*pks27* strains with *B. amyloliquefaciens* did not differ significantly (Figure 3A,B).

In confrontation with *A. nidulans*, 116 SM genes present in 36 SMBGCs of *C. graminicola* were transcriptionally de‐regulated (Figures 4A and S4J). De‐regulated genes included 17 PKS, 3 NRPS and 4 PKS‐NRPS hybrid genes. Again, this finding highlights the specificity of the SM response, whereby 45 and 79 SM genes of *C. graminicola* were de‐regulated either in the confrontation with *B. amyloliquefaciens* or in that with *A. nidulans* , respectively, and only 37 SM genes were de‐regulated in both confrontations (Figures 2A, 4A and S4J). RT‐qPCR experiments confirmed de‐regulation of genes of clusters 27 and 35 of *C. graminicola* confronting *B. amyloliquefaciens* and *A. nidulans* , respectively (Figure S5A and S5C), and fully supported RNA‐Seq data.

When *B. amyloliquefaciens* is confronted with *C. graminicola* , 113 differentially regulated SM genes were identified in 11 SMBGCs, 60 of which showed increased and 53 decreased transcript abundances (Figures 2B, S4J, and Table S7). The de‐regulated genes included 8 NRPS in 11 clusters. SMBGCs 6 and 11, which are responsible for iturin and bacilysin formation, had 19 and 25 de‐regulated genes, respectively, across their entire clusters. The validity of the RNA‐Seq data was confirmed for two iturin (*ituB* and *ituC*) and three bacilysin biosynthesis genes (*bacA, bacC* and *bacE*) by RT‐qPCR analyses (Figure S5B).

In *A. nidulans* confronted with *C. graminicola* , 160 SM genes in 50 SMBGCs were de‐regulated, including 14 PKS, 14 NRPS, 1 PKS‐NRPS hybrid and 2 terpene synthase genes (Figures 4B, S4J and Table S7). Importantly, most genes of SMBGC 21, which is responsible for the production of the carcinogenic polyketide sterigmatocystin, showed increased transcript abundances in both RNA‐Seq and RT‐qPCR analyses (Figures 4B, cluster 21; S5D). Commercial sterigmatocystin, at the concentration used, caused only minor inhibition of vegetative hyphal growth of *C. graminicola* (Figure S2A; sterigmatocystin).

As a close link between oxidative stress and regulation of secondary metabolism is established in filamentous fungi (Montibus et al., 2015), we hypothesised that formation of toxic SMs may induce cell damage, oxidative stress and programmed cell death. Therefore, we analysed whether monooxygenase, oxidoreductase and/or other genes related to oxidative stress responses, reactive oxygen species metabolism or redox processes were de‐regulated in the confrontations studied here. Indeed, GO enrichment analyses highlighted that significant numbers of genes encoding monooxygenase, oxidoreductases, as well as FAD‐, heme‐ and iron‐binding proteins were de‐regulated (Figure S8).

Taken together, although both the bacterial and the fungal confrontation of *C. graminicola* resulted in distance inhibition, comparisons of genome‐wide transcriptional responses suggested specific recognition of different confrontation partners and translation into confrontation‐specific transcriptional SM responses.

### 3.3 The Confrontational Metabolome Reveals Formation of Putatively Toxic as Well as Fungicide‐Related Compounds in an Interaction‐Specific Manner

**FIGURE 5:**
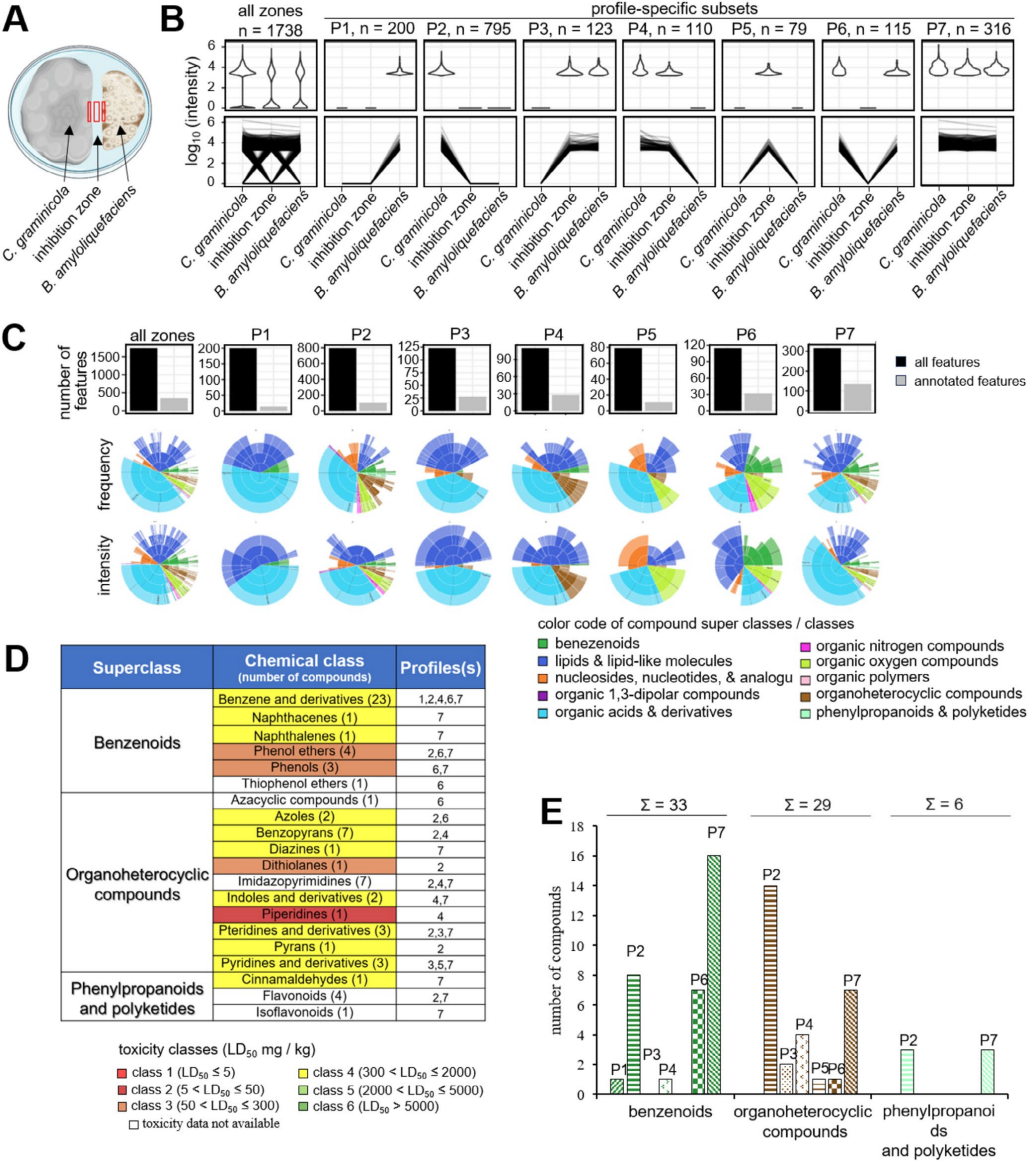
Metabolome analysis of *de novo* synthesised features in the *C. graminicola* – *B. amyloliquefaciens* confrontation. (A) Diagram of a Petri dish depicting the fungal‐bacterium confrontation. Red rectangles show metabolite sampling sites from *C. graminicola* and *B. amyloliquefaciens* colonies and from the inhibition zone. (B) The set of all detected features in all zones is separated into seven profile‐specific subsets (P1–P7). The profiles show intensities of features detected in confrontations in *B. amyloliquefaciens* alone (profile P1), or in *C. graminicola* alone (profile P2). Intensities of features present in *B. amyloliquefaciens* or *C. graminicola* and secreted into the medium are shown in profiles P3 and P4. Features present only in the confrontation zone are indicated as profile P5, and features present in *C. graminicola* and in the confrontation partner, but not in the inhibition zone are reflected by profile P6. Profile P7 indicates features present in *C. graminicola* , in the confrontation partner and in the inhibition zone. The upper panel shows violin plots revealing the distribution of measured compound intensities within individual samples. The lower panel represents line plots displaying the measured intensities per compound, where a black line links intensities corresponding to one feature over all samples. (C) Bar plots showing the number of total and annotated features (upper panel) and sunburst plots showing frequencies and intensities of annotated compound classes per set and sample. Four levels of compound annotation ranging from superclass, class, sub‐class and most specific class (inside to outside) are given. The colour code indicates compound superclasses. Fractions in the upper sunburst plots are based on the frequency (number of occurrences) and in the lower plot on the sum of measured intensities. (D) Number and occurrence of features belonging to the three major superclasses, including benzenoids, organoheterocyclic features and phenylpropanoids plus polyketides in profiles P1–P7. (E) Acute toxicity of features belonging to benzenoids, organoheterocyclic features and phenylpropanoids plus polyketides. Features are colour‐coded according to their toxicity classes. LD_50_ values were derived from ProTox 3.0 (Banerjee et al. 2024).

**FIGURE 6:**
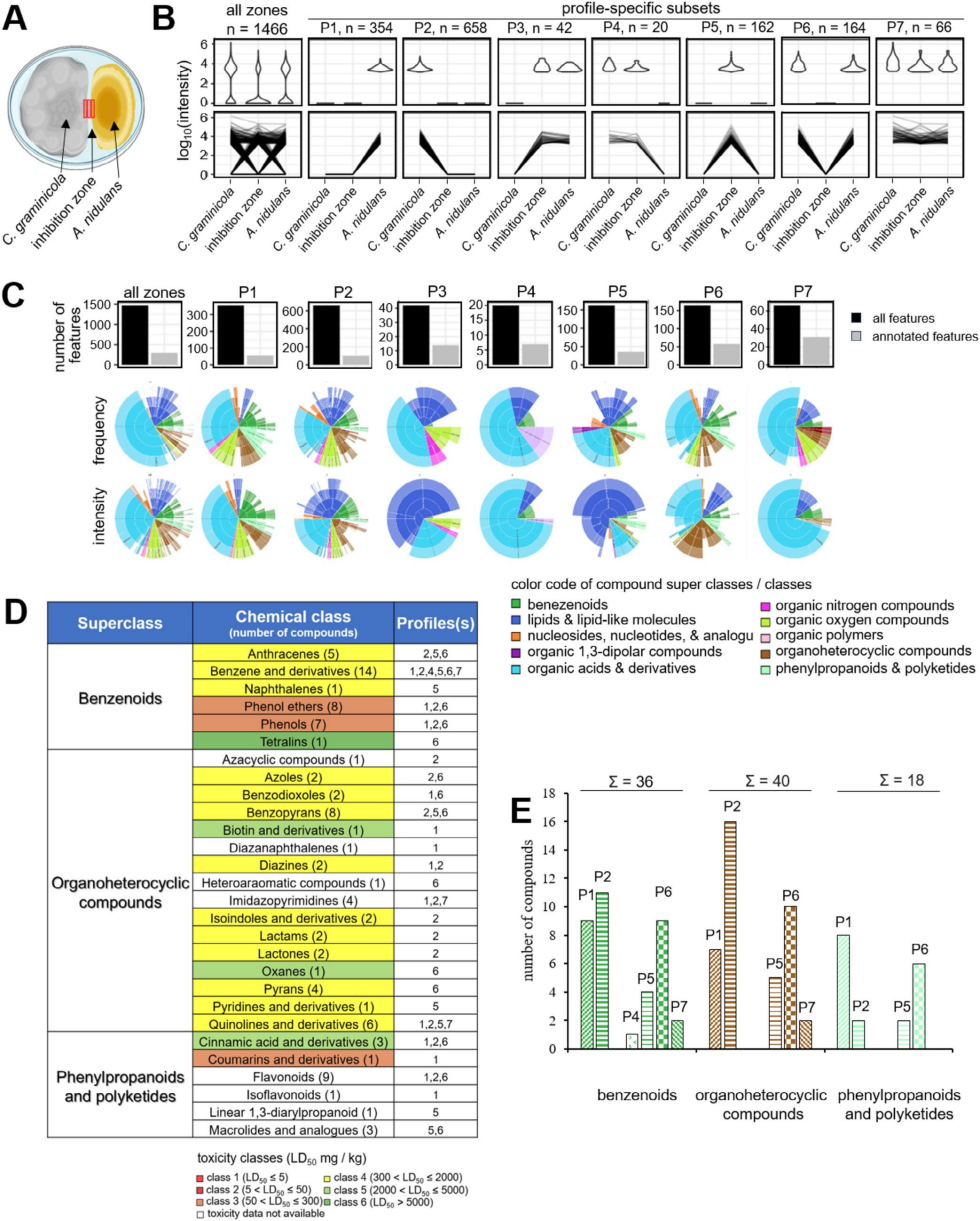
Metabolome analysis of features detected under *C. graminicola* vs. *A. nidulans* confrontation. (A) Diagram of a Petri dish depicting the fungal‐bacterium confrontation. Red rectangles show metabolite sampling sites from *C. graminicola* and *A. nidulans* colonies and from the inhibition zone. (B) The set of all detected features in all zones is separated into seven profile‐specific subsets (P1–P7). These profiles show intensities of features detected in confrontations in *A. nidulans* alone (profile P1), or in *C. graminicola* alone (profile P2). Intensities of features present in *A. nidulans* or *C. graminicola* and secreted into the medium are shown in profiles P3 and P4. Features present only in the confrontation zone are indicated as profile P5, and features present in *C. graminicola* and in the confrontation partner, but not in the inhibition zone are reflected by profile P6. Profile P7 indicates features present in *C. graminicola* , in the confrontation partner and in the inhibition zone. The upper panel shows violin plots revealing the distribution of measured compound intensities within individual samples. The upper panel shows violin plots revealing the distribution of measured compound intensities within individual samples. The lower panel represents line plots displaying the measured intensities per compound, where a black line links intensities corresponding to one compound over all samples. (C) Bar plots showing the number of total and annotated features (upper panel) and sunburst plots showing frequencies and intensities of annotated compound classes per set and sample. Four levels of compound annotation ranging from superclass, class, sub‐class and most specific class (inside to outside) are given. The colour code indicates compound superclasses. Fractions in the upper sunburst plots are based on the frequency (number of occurrences) and in the lower on the sum of measured intensities. (D) Number and occurrence of features belonging to the three major superclasses benzenoids, organoheterocyclic features and phenylpropanoids plus polyketides in profiles P1–P7. (E) Acute toxicity of features belonging to benzenoids, organoheterocyclic features and phenylpropanoids plus polyketides. Features are colour‐coded according to their toxicity classes. LD_50_ values were derived from ProTox 3.0 (Banerjee et al. 2024).

Alterations in SMBGC transcript abundances do not necessarily reflect alterations in SM concentrations. Therefore, to generate non‐targeted, high‐resolution compound partitioning data, we performed LC–MS/MS analyses of metabolites extracted from the edges of colonies of confrontation partners, as well as from the inhibition zone lacking fungal or bacterial cells (Figures 5A and 6A, red sections; Figure S9A,S9B, red sections). Metabolites extracted from the edges of the mycelia of solo‐cultures of each confrontation partner served as controls (Figure S9A, blue and yellow sections and Figure S9B, blue and brown sections). Surprisingly, 1738 and 1466 novel features were detected in the confrontations of *C. graminicola* with the biocontrol bacterium *B. amyloliquefaciens* and with the ubiquitous fungus *A. nidulans* , respectively (Figure S9C,S9D). Violin plots show the intensities and distribution of all features within individual samples, and of seven patterns of non‐overlapping feature distributions between the confrontation partners, referred to as profiles P1 to P7 (Figures 5B and 6B, all zones and P1–P7, upper panel, Tables S8 and S10). These patterns reflect the intensities of features detected in confrontations in *B. amyloliquefaciens* , in *A. nidulans* (Figures 5B and 6B; P1), or in *C. graminicola* alone (Figures 5B and 6B; P2). Intensities of features present in *B. amyloliquefaciens* , *A. nidulans* or *C. graminicola* and secreted into the medium are shown in profiles P3 and P4 (Figures 5B and 6B). Features present only in the confrontation zone are indicated as profile P5 in Figures 5B and 6B, and features present in *C. graminicola* and in the confrontation partner, but not in the inhibition zone are reflected by profiles P6 (Figures 5B and 6B). Profiles P7 indicate features present in *C. graminicola* , in the confrontation partners and in the inhibition zone (Figures 5B and 6B). Correspondingly, line plots indicate compound‐specific intensities, with intensities corresponding to one compound across samples linked by a black line (Figures 5B and 6B, all zones and P1–P7, lower panel). Intriguingly, profiles P3–P5, exhibiting secreted molecules, represent 312 features in the *C. graminicola* – *B. amyloliquefaciens* and 224 features in the *C. graminicola* – *A. nidulans* confrontation. In addition, profiles P7 of the two confrontations show very distinct numbers of features, that is 316 in the fungus–bacterium and only 66 in the fungus–fungus confrontation (Figures 5B, 6B, 5C and 6C, upper panel). Importantly, in both confrontations and in all profiles only a minor fraction of features is annotated (Figures 5C and 6C, upper panel).

Applying the software tool Canopus (https://bio.informatik.uni‐jena.de/software/canopus/) and the chemical ontology system ClassyFire (Djoumbou Feunang et al. 2016) allowed annotation of some of the SM features to chemical compound superclasses and classes. The fractions of annotated compound classes per profile‐specific subset and sample have been used to generate Sunburst plots, giving rise to four compound specificity levels, that is superclasses, classes, subclasses and most specific subclasses, with colour‐coded annotated compound superclasses (Figures 5C and 6C; inside to outside, lower panels). Fractions in the upper and lower Sunburst plot panels are based on frequencies of occurrence (frequency) or the sum of measured intensities (intensity) of features, respectively. In the *C. graminicola* – *B. amyloliquefaciens* confrontation, of a total of 68 compounds, 33 fell into 6 classes of the superclass of benzenoids, 29 belonged to 11 classes of organoheterocyclic compounds and 6 belonged to three classes of phenylpropanoids and polyketides (Figure 5D; Table S9), with distinct compounds attributed to different profiles (Figure 5E). In the *C. graminicola* – *A. nidulans* confrontation, the numbers of chemistries and classes they belong to were more complex, with 94 newly synthesised compounds identified. Six, 16 and six classes were members of the superclasses of benzenoids, organoheterocyclic compounds, and phenylpropanoids and polyketides, respectively (Figure 6D; Table S11), and were present in distinct profiles (Figure 6E). Interestingly, compound classes such as piperidines and cinnamaldehydes were newly synthesised only in the *C. graminicola* – *B. amyloliquefaciens* confrontation, whereas azoles were discovered in both confrontations. In fact, these three classes of chemicals represent lead structures of synthetic fungicides, possibly explaining the establishment of inhibition zones.

As an approximation of the toxicological potential of chemistry classes to mammals, toxicities of their lead structures were subjected to computational toxicity estimations, using ProTox 3.0 (https://tox.charite.de/protox3/index.php?site=home; Banerjee et al. 2024). These algorithms employ a comprehensive database of approximately 40,000 compounds with known LD_50_ values from rodent experiments (Banerjee et al. 2024). Intriguingly, MS/MS analyses of chemistries produced in the *C. graminicola* – *A. nidulans* confrontation yielded feature_562_363.19s_426.13106 Da, with a retention time (RT) of 363.19 s and an m/z value of 427.14. Four fragments with m/z means of 247.093, 381.131, 391.118 and 409.127 were identified. Annotation using the MetFrag software (Wolf et al. 2010) suggests that this compound is an isoflavonoid (Figure 6D, isoflavonoid, profile P1), that is the rotenoid villosinol (Figures S10), that has so far been identified in roots of the plant *Tephrosia villosa* (Muiva‐Mutisya et al. 2014). However, it is important to emphasise that predicted acute toxicities of molecules produced during confrontations, as deduced from lead structures, may differ significantly from actual toxicities of specific compounds. Thus, although a toxicity class prediction is not provided for the chemical class of isoflavonoids, ProTox 3.0 estimates an acute toxicity of 4 mg/kg body weight for villosinol. This significant toxicity is plausible, as rotenoids act as mitochondrial respiratory inhibitors that interfere with the transfer of electrons from iron–sulphur centres of complex I to ubiquinone (https://pubchem.ncbi.nlm.nih.gov/compound/Rotenone).

The Venn diagram shown in Figure S11A and the corresponding Table S12 indicate that only 282 newly formed features were common to both the *C. graminicola* – *B. amyloliquefaciens* and the *C. graminicola* – *A. nidulans* confrontation. As an indication of the high specificity of the metabolic response in the distinct confrontations analysed, 1456 and 1185 novel features were only detected in either the fungus–bacterium or in the fungus–fungus confrontation. Alluvial plots of the 282 common features indicated that some of these common features occur in different profiles, depending on the confrontation, and underline the plasticity of confrontation‐specific metabolic responses (Figure S11B). For example, of the 153 features newly formed in the mycelium of *C. graminicola* confronting *B. amyloliquefaciens* (Figure S11B; *C.g*. vs. *B.a*., profile P2), 132 were also present in the mycelium of this fungus when it confronted *A. nidulans* (Figure S11B; *C.g*. vs. *A.n*., profile P2). Only a small fraction of the 153 features of *C. graminicola* discovered in the bacterial confrontation was secreted (Figure S10B; *C.g*. vs. *B.a*., profile P5) or present in both partners in the *C. graminicola* – *A. nidulans* confrontation (Figure S11B; *C.g*. vs. *A.n*., profile P6).

Non‐targeted, high‐resolution LC–MS/MS analyses not only unravelled the magnitude of SM responses in distinct microbial confrontations but also allowed to functionally address the role of the core Pks of SMBGC 27 in the *C. graminicola* – *B. amyloliquefaciens* confrontation. To elucidate the reason for the lack of an effect of the *PKS27* deletion on the width of the inhibition zone (Figures 3A,B), we extracted metabolites from *C. graminicola* WT and the Δ*pks27* strains either growing in solo‐cultures or in confrontations. Surprisingly, while PCAs revealed a clear distinction of the WT and the ectopic strain from the Δ*pks27* strains in solo‐cultures, all strains were very similar during confrontations with *B. amyloliquefaciens* (Figure 3C). Moreover, in solo‐cultures LC–MS/MS analyses indicated some 3000 features in WT and the ectopic strain, but more than 5100 features in the Δ*pks27* mutants (Figure 3D), suggesting that the product(s) of SMBGC 27 may indirectly or directly repress compound formation. In solo‐cultures, 2401 common features were detected in both the WT and the Δ*pks27* strains, and more than 2700 features were exclusively detected in the Δ*pks27* mutant (Figure 3E). In the *C. graminicola* – *B. amyloliquefaciens* confrontation, however, feature numbers derived from LC–MS/MS studies were strongly increased in WT and ectopic strains but remained largely unaltered in the Δ*pks27* strains (Figure 3F). Unexpectedly, the shift in shared features from 2401 in solo‐cultures of WT and Δ*pks27* to 3919 in confrontations, along with the concomitant reduction of Δ*pks27*‐specific feature numbers from 2726 to 810 (Figure 3G), does not support the hypothesis that SMBGC 27 product(s) contribute to repression of gene expression. In line with feature numbers, annotated SMs such as benzenoids, organoheterocyclic compounds and phenylpropanoids/polyketides were increased in solo‐cultures of the Δ*pks27* strains, when compared with the solo‐cultures of WT and the ectopic strain. Again, very similar numbers of these compounds were detected in confrontations of WT, the ectopic and the Δ*pks27* strains with the biocontrol bacterium (Figure 3H). Apparently, despite the deletion in *PKS27* causing a de‐repression in feature‐synthesis in solo‐cultures, this effect was lost during confrontations. Currently, we have no explanation for this effect, but the data strongly suggest a new level of transcriptional control of the genes whose products are responsible for the synthesis of these compounds.

Collectively, the data presented here suggest that activation of SMBGCs and synthesis of molecules are primarily confrontation‐specific. Non‐targeted, high‐resolution LC–MS/MS analyses and feature annotations, as based on the current state of the databases used, suggest that several of the features identified in the confrontations may exhibit significant acute and unknown chronic toxicities in mammals.

## 4 Discussion

The detrimental toxicity of fungal SMs has harmed humanity for centuries (Lee 2009; Pitt and Miller 2017). Today, more than 300 mycotoxins, including ochratoxin A, fumonisins, trichothecenes, zearalenone, aflatoxin and sterigmatocystin, are of serious concern to human health, primarily due to their enormous carcinogenic potential (Brown et al. 2021; Esheli et al. 2022; Pickova et al. 2021; Sun et al. 2023; Wu 2014; Xu 2022; Zingales et al. 2020). Collectively, in fungi, more than 15,000 distinct SMs have been identified (Zhgun 2023), yet only in a few cases are the signals that trigger activation of SMBGCs and synthesis of their products known (Brakhage and Schroeckh 2011; Keller 2019; König et al. 2013; Schroeckh et al. 2009). Among the few examples of signals affecting SM synthesis are arginine‐derived polyketides of *Streptomyces* species. These arginoketides mediate induction of SM formation in fungi belonging to the genera *Aspergillus* and *Penicillium* in a cross‐kingdom fashion. The SMs formed in response to arginoketides are thought to induce a secondary wave of SM production, potentially shaping the broader structure and function of soil microbial communities (Krespach et al. 2023). Indeed, several putatively toxic SMs and their targets in microbial confrontation partners have been described, and the fact that successful defence against competing microbes is primarily chemistry‐based is meanwhile widely accepted (Brakhage 2013; Künzler 2018; Macheleidt et al. 2016). One of the interesting aspects of our study was that in the interaction between the maize pathogen and *B. amyloliquefaciens* , the biocontrol bacterium produced compounds such as iturin A and bacilysin targeting fungal cell wall biosynthesis in an inter‐kingdom confrontation, as indicated by formation of large hyphal swellings in confrontations. Likewise, in the bacterium–fungus, but not in the fungus–fungus confrontation, formation of another cell wall‐targeting compound, that is a cinnamaldehyde, was discovered. In vitro studies employing baker's yeast indicated that trans‐cinnamaldehyde is a non‐competitive inhibitor of *β*‐1,3‐glucan synthase and an inhibitor of chitin synthase 1 (Bang et al. 2000) and, moreover, showed anti‐aflatoxigenic effects in *A. flavus* (Wang et al. 2019). Further highlighting the specificity of SM responses in the distinct confrontations, almost all genes of SMBGC27 in *C. graminicola* are activated when confronting *B. amyloliquefaciens* , but not in confrontations with *A. nidulans* . Moreover, SMBGC21 of *A. nidulans* , which is responsible for sterigmatocystin formation, is activated in response to *C. graminicola* . This SM, like the closely related aflatoxin, is mutagenic not only in mammalian, but also in microbial cells (Csenk et al. 2022), and its formation may provide an advantage in confrontations. In spite of the acceptance of the predominant role of chemicals in interactions, questions regarding the complexity, plasticity and specificity of microbial chemical defence remain largely unanswered (Künzler 2018).

The hypothetical complexity of the reservoirs of distinct fungal and bacterial compounds has been highlighted by comparative genomic and cheminformatic analyses, employing a set of 1037 genomes from species across the fungal kingdom, as well as of 5453 bacterial genomes (Robey et al. 2021). Remarkably, more than 36,399 fungal SMBGCs are organised into 12,067 gene‐cluster families, and anchoring these cluster families with reference to SMBGCs has allowed annotation of more than 2000 clusters with predicted metabolite scaffolds. In total, more than 15,000 fungal and over 9000 bacterial compounds have been identified and reveal a landscape of largely species‐specific compound reservoirs (Robey et al. 2021).

As a large number of these compounds synthesised in microbial communities may be putatively toxic SMs (Netzker et al. 2015), it is surprising that distance inhibition was not observed to occur in microbial populations on maize leaves imprinted onto agar plates, as indicated by the lack of inhibition zones between colonies of numerous distinct species shown in this work. Likewise, it is surprising that a screen of more than 700 environmental isolates from apple trees and fruits identified only four bacterial and three fungal isolates capable of reducing the development of fruit‐rot lesions caused by the apple pathogens *Pezicula malicorticis* and *Nectria galligena* (Schiewe and Mendgen 1992). Over the last decades, strategies have been developed to increase the efficacies to identify novel microbial biocontrol agents, as described in detail in a recent excellent review (Collinge et al. 2022, and references therein). It is not surprising, however, that the commercially available antagonist *B. amyloliquefaciens* and one of the best studied SM producers, *A. nidulans* , displayed distance inhibition in confrontations with *C. graminicola* . Employing combined transcriptome and metabolome data, our studies revealed an enormous plasticity and specificity in the synthesis of complex arrays of novel SMs synthesised in the confrontations of *C. graminicola* with *B. amyloliquefaciens* or *A. nidulans* . Interestingly, many genes of the majority of the SMBGCs were de‐regulated in all partners in both confrontations studied. However, only in a few cases, for example in SMBGC27 of *C. graminicola* in response to *B. amyloliquefaciens* , almost all genes of the cluster showed increased transcript abundances. SMBGC27 does not harbour a gene encoding a transcription factor, leaving the mechanism leading to largely uniform activation open. As indicated above, also the function of the product of SMBGC27 remains elusive. Increased compound numbers in Δ*pks27* deletion strains suggest that the product of SMBGC27 appears to function as a repressor of several genes, including SM genes, in solo‐cultures. However, as the cluster is strongly activated during confrontation with *B. amyloliquefaciens* , the expected repressor function appears inconsistent with the observed increase in SM production. One may hypothesise that the repressor function is overridden by another as yet unidentified regulatory factor(s).

An important discovery was that several compound classes identified in these confrontations, for example azoles, piperidines and cinnamaldehydes, exhibit structural similarities with lead structures of synthetic fungicides used in plant disease control (FRAC codes 3, 5 and BM03). Moreover, rotenone, a compound with high similarity to the rotenoid villosinol putatively annotated in the *C. graminicola* – *A. nidulans* confrontation, has been used as an insecticide and acaricide (IRAC code 21) for many years. Thus, in several cases, secondary metabolites synthesised in microbial confrontations closely resemble commercially available pesticides and may thus address the same target site(s) when synthesised in confrontations with biological control agents.

The major concern, however, relates to the carcinogenic activity of microbial SMs. For example, after ingestion, the polyketide‐mycotoxins aflatoxin and sterigmatocystin are oxidised by cytochrome P450 enzymes, yielding C‐8,9‐epoxides, which form covalent bonds between C8 of the toxin and N7 of guanine bases of DNA. Replication of DNA containing these adducts causes G → T transversions, and indeed, high frequencies of this mutation at codon 249 of the tumour suppressor gene p53 have been reported in hepatocellular carcinomas from populations exposed to aflatoxin‐contaminated food in South Africa and China (Wang and Groopman 1999). Hepatocellular carcinomas were also observed in rats exposed to tetralin or anthracene (NationalToxicologyProgram 2011; Takeda et al. 2023). Compounds belonging to these classes were identified in the *C. graminicola* – *A. nidulans* confrontation studied here. Moreover, the insecticidal rotenone induced neoplastic, paraneoplastic and preneoplastic lesions in rats and is thus considered an environmental carcinogen (Gosálvez 1983). It is possible that villosinol may also exhibit comparable activities. In the light of the large feature numbers observed in the fungus–bacterium and in the fungus–fungus interactions studied here, synergistic activities of toxic compounds in carcinogenesis may be of high relevance. Notably, mice co‐colonised with colibactin‐producing *Escherichia coli* and enterotoxigenic *Bacteroides fragilis* showed increased DNA damage, faster colon tumour onset and greater mortality than mice colonised by either bacterial strain alone (Dejea et al. 2018). Moreover, several of the compounds assigned to different chemical classes may putatively show acute toxicity, as deduced from the LD_50_ values assigned to the class by computational toxicity estimations. In confrontations between *C. graminicola* and *B. amyloliquefaciens* , one piperidine compound was identified to be produced and secreted by the fungal partner and assigned to toxicity class 2, with a putative LD_50_ value between 5 and 50 mg/kg body weight. In addition, seven benzenoids and one organoheterocyclic compound were found and assigned to toxicity class 3. In the confrontation between *C. graminicola* and *A. nidulans* , 16 compounds belonging to the benzenoids and phenylpropanoids/polyketides are putative class 3 toxicity members. Compounds of toxicity class 2 are regarded as highly toxic, whereas members of toxicity class 3 are considered moderately toxic (Erhirhie et al. 2018). The acute risk posed by mixtures of substances is difficult to assess (Bloch et al. 2023). Although only some of the compounds identified in fungus–bacterium and fungus–fungus confrontations exhibited relevant toxicities, the toxicity of complex compound mixtures may hold unknown risks for consumers.

Our studies demonstrate that a large array of novel and highly diverse molecules are synthesised in the confrontations between the maize pathogen *C. graminicola* and the biocontrol bacterium *B. amyloliquefaciens* or in confrontations with *A. nidulans* . The confrontation specificity of the arsenal of chemistries employed may not be surprising, given the long period of chemical evolution in fungus–bacterium and fungus–fungus interactions. Indeed, fungal confrontations have been uncovered in excellently preserved 400‐million‐year‐old mycoflora samples of the Lower Devonian Rhynie chert ecosystem (Hass et al. 1994). Thus, as the co‐evolution of plant‐associated microorganisms with each other is well documented, it is not surprising that in the two microbial confrontations studied here, 1738 and 1466 features were newly synthesised. These compounds are confrontation‐specific and are not synthesised in solo‐cultures of the confrontation partners.

Plausibly, confrontation‐specific compounds could not be evaluated in legislative efforts for antagonists to be used in biological plant protection. In comparison, defined synthetic compounds submitted for approval as plant protection products are intensively evaluated and peer‐reviewed by the member states and the European Food Safety Authority. Approval criteria defined in Regulation (EC) No 1107/2009 (https://eur‐lex.europa.eu/eli/reg/2009/1107/oj) exclude candidate compounds suspected to display carcinogenic or otherwise adverse activities. It is conceivable, however, that stress imposed by application of fungicides, which are neither toxic nor carcinogenic themselves, may activate SMBGCs in target fungi when applied at sublethal concentrations. In order to investigate genome‐wide transcriptional responses, including the responses of SMBGC genes, to sublethal fungicide stress, the wheat head blight fungus *Fusarium graminearum* was treated with 5 ppm concentrations of the azole fungicide Tebuconazole (Eisermann 2023). Importantly, even at a low threshold of log2FC ≥ 1, only 14 and 4 de‐regulated SM genes were identified in two independent experiments (Iris Eisermann, personal communication). For comparison, in *C. graminicola* confronting *B. amyloliquefaciens* or *A. nidulans* , 82 and 116 SMBGC genes were de‐regulated, respectively. The comparison of transcriptome data clearly supports the idea that synthesis of putatively toxic SMs is more strongly stimulated in microbial confrontations, as compared to stress exerted by fungicide challenge. For further comparisons between SM responses to confronting microorganisms and fungicides, it would be interesting to have high‐resolution metabolome data available for both stresses.

Collectively, combinations of transcriptome and metabolome analyses of a plant pathogenic fungus with a biocontrol bacterium or a model fungus for SM analyses have revealed dramatic reprogramming in microbial SMBGCs and synthesis of confrontation‐specific compounds that may exert significant putative health risks to consumers. The fact that a large array of novel compounds with unknown acutely toxic and/or carcinogenic properties were formed in a confrontation between an approved biocontrol bacterium and a plant pathogenic fungus calls into question whether biological disease control truly promotes consumer safety. These data demand more stringent analyses of microbial confrontation‐induced SMs before the approval of microorganisms for plant protection.

## 5 Conclusions

In spite of the enormous risk posed by microbial toxins, in public risk perception pesticides pose a greater hazard (Muri et al. 2009; Williams and Hammitt 2001). Therefore, following societal and political demands, microbial biological control agents are proposed for use to compensate for the reduction of approved synthetic fungicides (Beckerman et al. 2023; Oliveira‐Garcia et al. 2021). However, although a large body of literature indicates that microbial confrontations are decided based on the toxicity of chemicals formed (Künzler 2018, and references therein), synthesis of putatively consumer‐toxic compounds in confrontations between biological control agents and plant pathogenic fungi is poorly investigated. Our work shows that secondary metabolism is severely altered in confrontations of the maize pathogen *Colletotrichum graminicola* with the approved biocontrol bacterium *B. amyloliquefaciens* and the ubiquitous fungus *A. nidulans* . Surprisingly, high‐resolution LC–MS/MS revealed a large repertoire of novel features synthesised in confrontations, several of which share lead structures with synthetic fungicides. These discoveries shed light on the mode by which antagonists inhibit disease development in crops. In addition, several compounds generated in confrontations belong to chemical classes harbouring toxic and/or carcinogenic substances. Dual confrontations as studied here show an enormous chemical complexity. However, the large number of confrontations established by applying antagonistic microorganisms to the densely colonised crop phylloplane (Leveau 2019; Vorholt 2012) will likely result in the establishment of more confrontations and a more complex chemical environment, as compared to dual confrontations. One may thus assume that our study significantly underestimates the chemical complexity that may be formed in agro‐environments treated with biocontrol agents. We have shown that the vast majority of compounds formed are confrontation‐specific, making the number, the chemical structure and the toxicity of compounds in antagonist‐treated crops difficult to predict (Bloch et al. 2023; Deising et al. 2017).

## Author Contributions

**Bennet Rohan Fernando Devasahayam:** conceptualization, methodology, formal analysis, writing – original draft, writing – review and editing, data curation. **Henriette Uthe:** methodology, formal analysis, writing – review and editing, data curation. **Yvonne Poeschl:** methodology, formal analysis, writing – review and editing, data curation. **Holger B. Deising:** conceptualization, funding acquisition, supervision, writing – original draft, writing – review and editing, data curation.

## Conflicts of Interest

The authors declare no conflicts of interest.

## Supporting information


**FIGURE S1** Split plate assays indicate that volatile organic compounds (VOCs) do not contribute to distance inhibition. Note that colonies growing in monocultures in different compartments show comparable distances to the split as neighbouring colonies separated by the split.
**FIGURE S2**. Growth inhibition of *C. graminicola* by Iturin A and Sterigmatocystin measured by Kirby‐Bauer disc diffusion assays. (A) Left panel Petri dishes with colonies of *C. graminicola* and a filter disc (arrow) containing Iturin A or Sterigmatocystin (1 mg/mL). Ethanol and/or methanol acts as a solvent control. Arrowheads indicate inhibition zones. The asterisk indicates areas of reduced conidiation. Photographs were taken at 12 dpi. White rectangles mark the area from which samples were taken for microscopy. Arrowheads in Differential interference (DIC), Calcofluor White and in merged micrographs indicate hyphal swellings. Scale bar corresponds to 50 μm. (B) Halo area indicative of hyphal growth inhibition increased with increasing Iturin A concentrations. Data are means of three independent biological replicates. Error bars are +SDs.
**FIGURE S3**. Quantitative assessment of hyphal protrusions in *C. graminicola* in confrontation with *B. amyloliquefaciens* . (A) Schematic illustration of the confrontation zone between *C. graminicola* and *B. amyloliquefaciens* , with coloured rectangles indicating sampling sites at increasing distances of the fungal hyphae from the bacterial colony. (B) Percentage of the area covered by hyphal protrusions at different distances from the colony edge of *C. graminicola* . Error bars are +SDs.
**FIGURE S4**. Transcriptome analysis of differentially expressed genes (DEGs) under confrontations. (A) Principal component analyses (PCA) show clear cluster separation of *C. graminicola* solo‐cultures and hyphae confronting *B. amyloliquefaciens* (*Cg* vs. *Ba*) or *A. nidulans* (*Cg* vs. *An*). (B) PCA plot showing distinctness of *B. amyloliquefaciens* monocultures and cultures confronting *C. graminicola* (*Ba* vs. *Cg*). (C) PCA plot showing distinctness of monocultures of *A. nidulans* and mycelia confronting *C. graminicola* (*An* vs. *Cg*). Sample groups are indicated by different colour codes. Each replicate is plotted as an individual data point. (D) Heatmap of the sample‐to‐sample distance matrix obtained from monoculture of *C. graminicola* and confrontations with *B. amyloliquefaciens* and *A. nidulans*. (E) Heatmap of the sample‐to‐sample distance matrix of *B. amyloliquefaciens* monocultures and cultures confronting *C. graminicola*. (F) Heatmap of the sample‐to‐sample distance matrix of *A. nidulans* monocultures and cultures confronting *C. graminicola*. The colour codes in sub‐figure (D–F) indicate the distance between the samples, as based on the Z‐score. Dark blue denotes shorter distance, that is replicates are grouped closer in distance. (G) DEGs identified in different confrontations and confrontation partners. Red and blue bars indicate increased (FC > 2) and decreased (FC < 0.5) transcript abundances with adjusted *p*‐value < 0.05. (H) Venn diagram representing the distribution of the DEGs of *C. graminicola* confronting *B. amyloliquefaciens* (*Cg* vs. *Ba*) or *A. nidulans* (*Cg* vs. *An*). The number in the overlap denotes the mutual DEGs between distinct confrontations. (I) Scatter plot showing confrontation‐specific and shared DEGs of *C. graminicola* . Genes are grouped according to functional categories. (J) Bar graph showing the number of SM genes de‐regulated in different confrontation partners in distinct confrontations. Red and blue bars indicate increased and decreased transcript abundances of differentially expressed SM genes.
**FIGURE S5**. Validation of RNA‐Seq data by reverse transcription‐quantitative polymerase chain reaction (RT‐qPCR) as an independent method. (A) Seventeen genes of SMBGC 27 of *C. graminicola* showing increased transcript abundances in confrontation with *B. amyloliquefaciens* were validated by RT‐qPCR. (B) As for the RNA‐Seq analyses, two iturin and three bacilysin genes of SMBGCs 6 and 11 in *B. amyloliquefaciens* showed increased transcript abundances in confrontation with *C. graminicola* . (C) Confirmation of increased transcript abundances of the five genes of SMBGC35 of *C. graminicola* confronting *A. nidulans* . (D) Twenty out of 24 genes of the sterigmatocystin genes of SMBGC 21 of *A. nidulans* exhibited increased transcript abundances also when analysed by RT‐qPCR.
The physical maps of the corresponding SMBGCs and genes with increased transcript abundances, as analysed by RNA‐Seq studies, are given above the respective bar plot. The constitutively expressed actin biosynthesis genes of *C. graminicola* and *A. nidulans* , as well as the *gyrA* gene of *B. amyloliquefaciens* served as standards. Data shown are the means of three independent biological replicates and three technical replicates. Error bars are +SDs.
**FIGURE S6**. Targeted deletion of *PKS27* of *C. graminicola* . (A) *PKS27* of SMBGC 27, encoding a polyketide synthase, was replaced by a construct consisting of the hygromycin phosphotransferase gene *hph* of *E. coli* and the 5′‐and 3′‐flanks of *PKS27* of *C. graminicola* . *Bam*HI sites and size of DNA fragments are indicated. The position of the probe is indicated as a black line over the 5′‐flank. (B) Genomic Southern blot of *Bam*HI‐digested genomic DNA of the wildtype (WT), an ectopic (ect.) and two Δ*pks27* strains. The blot was hybridised with the 5′‐flank‐specific probe.
**FIGURE S7**. *PKS27* of *C. graminicola* is not required for vegetative growth, conidiation or virulence. The colony phenotype (A) as well of radial growth rates (B) of WT, ectopic (ect.) and ∆*pks27* strains grown on PDA are not discernible. Photographs in (A) were taken at 14 dpi. Growth rates (B) were measured daily. (C) WT, ectopic (ect.) and ∆*pks27* strains had formed comparable numbers of conidia onto PDA at 14 dpi. (D) The shape of the conidia was not altered by deletion of *PKS27*, but (E) the length of conidia of ∆*pks27* strains was marginally reduced. (F) The percentage of appressoria differentiated from falcate conidia on maize (cv. Mikado) leaf surfaces was slightly but statistically significantly (*p* ≤ 0.05) higher in ∆*pks27* strains, but (G) appressoria (white arrowheads) of both WT and ∆*pks27* strains invaded the host epidermal cells and formed normal biotrophic hyphae (black arrowheads). (H) WT, ectopic (ect.) and ∆*pks27* strains elicited comparable disease symptoms.
**FIGURE S8**. Gene Ontology (GO) enrichment analysis of differentially expressed genes (DEGs) under microbial confrontations. Bubble plots display enriched GO molecular function terms among DEGs identified during pairwise microbial confrontations. The X‐axis represents the gene ratio, defined as the number of DEGs associated with a GO term divided by the total number of genes annotated to that term. Bubble sizes indicate the number of DEGs mapped to each GO term. Bubble colour corresponds to statistical significance, represented as the negative log10 of the adjusted *p*‐value. Only GO terms with adjusted *p*‐values less than 0.05 are included.
**FIGURE S9**. Sample collection for metabolome analyses and Venn diagram showing numbers of chemistries newly synthesised in distinct confrontations. (A and B) Samples collected from 5 mm of the confronting culture margins and from the area between cultures lacking fungal or bacterial cells (red rectangles). Samples from margins of monocultures (blue, yellow and brown marked areas) served as controls. Blue and yellow areas denote the sample isolation spots in solo‐cultures, and red area in co‐culture denotes the sample isolation from confrontation partners and inhibition zone. (C and D) Venn diagram showing 1738 molecules specifically synthesised in the confrontation between *C. graminicola* and *B. amyloliquefaciens* , and 1466 in the *C. graminicola* vs. *A. nidulans* confrontation.
**FIGURE S10**. Annotation of compound feature 562 from the *C. graminicola* – *A. nidulans* confrontation using MetFrag software. The compound is produced in hyphae of *A. nidulans* (see Figure 5D, isoflavonoids, profile 1) and was annotated as villosinol. The MS/MS spectra, with a retention time (RT) of 363.19 s and an m/z value of 427.14, revealed four characteristic fragments (m/z 247.093, 381.131, 391.118 and 409.127). These fragments correspond to the stepwise loss of chemical groups from the villosinol molecule. The structures of these fragments are illustrated alongside the MS/MS spectrum.
**FIGURE S11**. Confrontation‐ and profile‐specificity of features synthesised in the confrontations of *C. graminicola* with *B. amyloliquefaciens* or *A. nidulans* . (A) Venn diagram indicating that of the 1738 and 1466 features synthesised in the fungus–bacterium and in the fungus–fungus confrontation, respectively. Only 282 were common to both interactions. (B) Alluvial plot showing comparison of the 282 features shared between both confrontations. Profiles P1–P7 are as in Figures 5 and 6. Stacked bar plots for each of the confrontations show the number of features per subset, with colours corresponding to profile plots. Lines connect the same individual features.

**TABLE S1.** DEGs of *C. graminicola* expressed in confrontation with *B. amyloliquefaciens* .
**TABLE S2**. DEGs of *B. amyloliquefaciens* expressed in confrontation with *C. graminicola* .
**TABLE S3**. DEGs of *C. graminicola* expressed in confrontation with *A. nidulans* .
**TABLE S4**. DEGs of *A. nidulans* expressed in confrontation with *C. graminicola* .
**TABLE S5**. List of specific and shared DEGs from *C. graminicola* produced in different confrontations.
**TABLE S6**. Confrontation‐specific and shared DEGs of different gene categories of *C. graminicola* .
**TABLE S7**. SM cluster genes of *C. graminicola* , *B. amyloliquefaciens* and *A. nidulans* differentially expressed in confrontations.
**TABLE S8**. Features obtained from three zones, that is the colony margin of *C. graminicola* , the colony margin of *B. amyloliquefaciens* and the inhibition zone.
**TABLE S9**. Acute toxicity of features belonging to benzenoids, organoheterocyclic features, and phenylpropanoids and polyketides formed in the in *C. graminicola* – *B. amyloliquefaciens* confrontation. Features are colour‐coded according to their toxicity classes, as indicated by their LD_50_ values.
**TABLE S10**. Features obtained from three zones, that is the colony margin of *C. graminicola* , the colony margin of *A. nidulans* and the inhibition zone.
**TABLE S11**. Acute toxicity of features belonging to benzenoids, organoheterocyclic features, and phenylpropanoids and polyketides in the *C. graminicola* – *A. nidulans* confrontation. Features are colour‐coded according to their toxicity classes, as indicated by their LD_50_ values.
**TABLE S12**. Profile‐specific feature intensities in the colony margin of *C. graminicola* , the colony margin of confrontation partners and in the inhibition zone.
**TABLE S13**. PCR primers used in this study.

## Data Availability

The data that supports the findings of this study are available in the supplementary material of this article.
