# Supplementary material for: Confrontations of the Pathogenic Fungus Colletotrichum graminicola With a Biocontrol Bacterium or a Ubiquitous Fungus Trigger Synthesis of Secondary Metabolites With Lead Structures of Synthetic Fungicides
Source: Environ Microbiol. 2025 Jul 14;27(7):e70145. doi: 10.1111/1462-2920.70145 (PMC12260340; doi:10.1111/1462-2920.70145)
Supplement: Supplementary file 1 — FIGURE S1 Split plate assays indicate that volatile organic compounds (VOCs) do not contribute to distance inhibition. Note that colonies growing in monocultures in different compartments show comparable distances to the split as neighbouring colonies separated by the split. FIGURE S2. Growth inhibition of C. graminicola by Iturin A and Sterigmatocystin measured by Kirby‐Bauer disc diffusion assays. (A) Left panel Petri dishes with colonies of C. graminicola and a filter disc (arrow) containing Iturin A or Sterigmatocystin (1 mg/mL). Ethanol and/or methanol acts as a solvent control. Arrowheads indicate inhibition zones. The asterisk indicates areas of reduced conidiation. Photographs were taken at 12 dpi. White rectangles mark the area from which samples were taken for microscopy. Arrowheads in Differential interference (DIC), Calcofluor White and in merged micrographs indicate hyphal swellings. Scale bar corresponds to 50 μm. (B) Halo area indicative of hyphal growth inhibition increased with increasing Iturin A concentrations. Data are means of three independent biological replicates. Error bars are +SDs. FIGURE S3. Quantitative assessment of hyphal protrusions in C. graminicola in confrontation with B. amyloliquefaciens . (A) Schematic illustration of the confrontation zone between C. graminicola and B. amyloliquefaciens , with coloured rectangles indicating sampling sites at increasing distances of the fungal hyphae from the bacterial colony. (B) Percentage of the area covered by hyphal protrusions at different distances from the colony edge of C. graminicola . Error bars are +SDs. FIGURE S4. Transcriptome analysis of differentially expressed genes (DEGs) under confrontations. (A) Principal component analyses (PCA) show clear cluster separation of C. graminicola solo‐cultures and hyphae confronting B. amyloliquefaciens (Cg vs. Ba) or A. nidulans (Cg vs. An). (B) PCA plot showing distinctness of B. amyloliquefaciens monocultures and cultures confronting C. grami [file EMI-27-e70145-s001.docx]

**Supplemental Material**

Confrontations of the pathogenic fungus *Colletotrichum graminicola* with a biocontrol bacterium or a ubiquitous fungus trigger synthesis of secondary metabolites with lead structures of synthetic fungicides

Bennet Rohan Fernando Devasahayam^1,2^, Henriette Uthe^2,3,5^, Yvonne Poeschl-Grau^2,4,5^, and Holger B. Deising^1,5,*^

^1^ Martin Luther University Halle-Wittenberg, Faculty of Nutritional Sciences III, Institute of Agricultural and Nutritional Sciences, Chair of Phytopathology and Plant Protection, Betty-Heimann-Str.3, D-06120 Halle / Saale, Germany

^2^ EcoMetEoR, Molecular Interaction Ecology, German Center for Integrative Biodiversity Research (iDiv) Halle-Jena-Leipzig, Puschstr. 4, D-04103 Leipzig, Germany

^3^ MetaCom, Leibniz Institute of Plant Biochemistry, Weinberg 3, Halle (Saale), 06120, Germany

^4^ Biometrics and Agricultural Informatics, Faculty of Natural Sciences III, Martin Luther University Halle-Wittenberg, Karl-Freiherr-von-Fritsch-Str. 4, D-06120 Halle / Saale, Germany

^5^ German Center for Integrative Biodiversity Research (iDiv) Halle-Jena-Leipzig, Puschstr. 4, D-04103 Leipzig, Germany

*Correspondence: [holger.deising@landw.uni-halle.de](mailto:holger.deising@landw.uni-halle.de)

**Content**

**Figure S1. Split plate assays indicate that volatile organic compounds (VOCs) do not contribute to distance inhibition.**

**Figure S2. Growth inhibition of *C. graminicola* by Iturin A and Sterigmatocystin measured by Kirby-Bauer disc diffusion assays.**

**Figure S3.** **Quantitative assessment of hyphal protrusions in *C. graminicola* in confrontation with *B. amyloliquefaciens*.**

**Figure S4. Transcriptome analysis of differentially expressed genes (DEGs) under confrontations.**

**Figure S5. Validation of RNA-Seq data by reverse transcription- quantitative polymerase chain reaction (RT-qPCR) as an independent method.**

**Figure S6. Targeted deletion of *PKS27* of *C. graminicola*.**

**Figure S7. *PKS27* of *C. graminicola* is not required for vegetative growth, conidiation or virulence.**

**Figure S8. Gene Ontology (GO) enrichment analysis of differentially expressed genes (DEGs) under microbial confrontations.**

**Figure S9. Sample collection for metabolome analyses and Venndiagram showing numbers of chemistries newly synthesized in distinct confrontations.**

**Figure S10. Annotation of compound feature 562 from the C. graminicola – A. nidulans confrontation using MetFrag software.**

**Figure S11. Confrontation- and profile-specificity of features synthesized in the confrontations of *C. graminicola* with *B. amyloliquefaciens* or *A. nidulans*.**

**Table S1. DEGs of *C. graminicola* expressed in confrontation with *B. amyloliquefaciens*.**

**Table S2. DEGs of *B. amyloliquefaciens* expressed in confrontation with *C. graminicola*.**

**Table S3. DEGs of *C. graminicola* expressed in confrontation with *A. nidulans*.**

**Table S4. DEGs of *A. nidulans* expressed in confrontation with *C. graminicola*.**

**Table S5. List of specific and shared DEGs from *C. graminicola* produced in different confrontations.**

**Table S6. Confrontation-specific and shared DEGs of different gene categories of *C. graminicola*.**

**Table S7. SM cluster genes of *C. graminicola*, *B. amyloliquefaciens*, and *A. nidulans* differentially expressed in confrontations.**

**Table S8. Features obtained from three zones, i.e. the colony margin of *C. graminicola*, the colony margin of *B. amyloliquefaciens*, and the inhibition zone.**

**Table S9. Acute toxicity of features belonging to benzenoids, organoheterocyclic features, and phenylpropanoids and polyketides formed in the in *C. graminicola* – *B. amyloliquefaciens* confrontation.** Features are color-coded according to their toxicity classes, as indicated by their LD_50_ values.

**Table S10. Features obtained from three zones, i.e. the colony margin of *C. graminicola*, the colony margin of *A. nidulans*, and the inhibition zone.**

**Table S11. Acute toxicity of features belonging to benzenoids, organoheterocyclic features, and phenylpropanoids and polyketides in the *C. graminicola* – *A. nidulans* confrontation.** Features are color-coded according to their toxicity classes, as indicated by their LD_50_ values.

**Table S12. Profile-specific feature intensities in the colony margin of *C. graminicola*, the colony margin of confrontation partners and in the inhibition zone.**

**Table S13. PCR primers used in this study.**


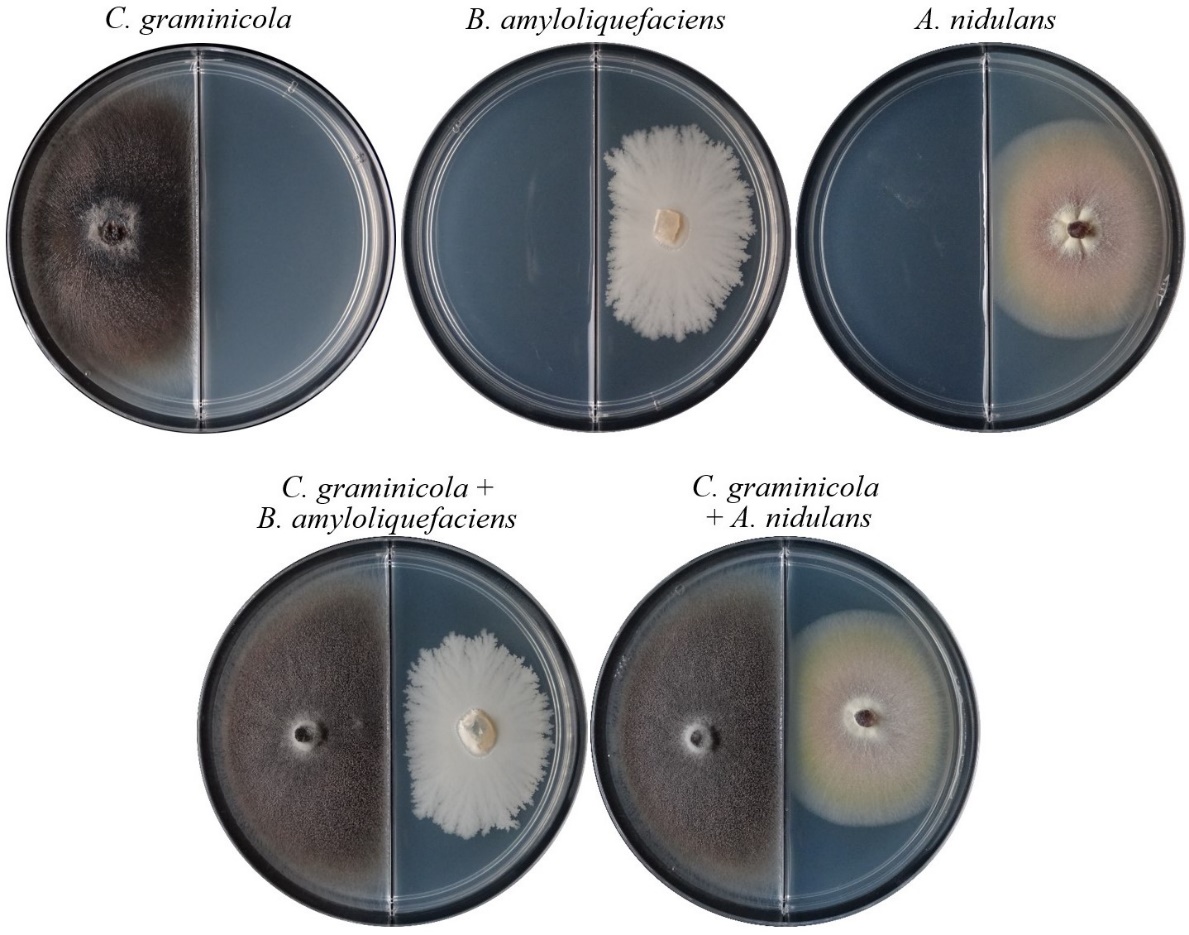


**Figure S1. Split plate assays indicate that volatile organic compounds (VOCs) do not contribute to distance inhibition.** Note that colonies growing in monocultures in different compartments show comparable distances to the split as neighboring colonies separated by the split.


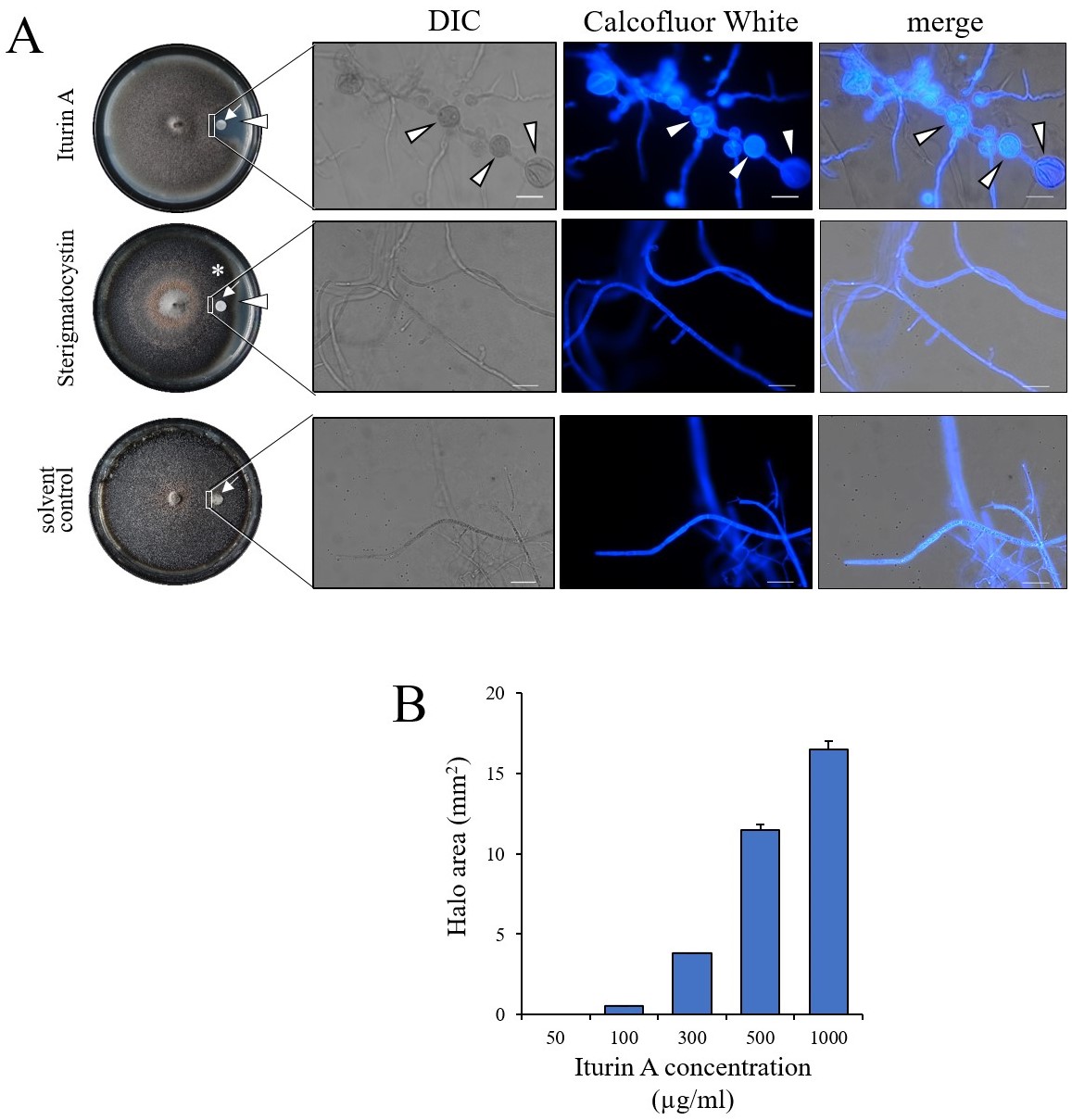


**Figure S2. Growth inhibition of *C. graminicola* by Iturin A and Sterigmatocystin measured by Kirby-Bauer disc diffusion assays.** (A) Left panel Petri dishes with colonies of *C. graminicola* and a filter disc (arrow) containing Iturin A or Sterigmatocystin (1mg/mL). Ethanol and/or methanol acts as a solvent control. Arrowheads indicate inhibition zones. The asterisk indicates areas of reduced conidiation. Photographs were taken at 12 dpi. White rectangles mark the area from which samples were taken for microscopy. Arrowheads in Differential interference (DIC), Calcofluor White and in merged micrographs indicate hyphal swellings. Scale bar corresponds to 50µm. (B) Halo area indicative of hyphal growth inhibition increased with increasing Iturin A concentrations. Data are means of three independent biological replicates. Error bars are +SDs.


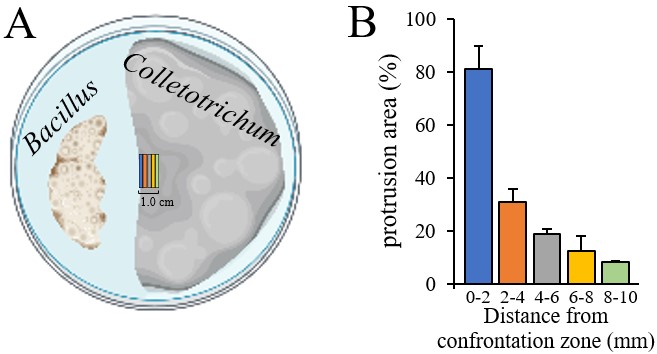


**Figure S3.** **Quantitative assessment of hyphal protrusions in *C. graminicola* in confrontation with *B. amyloliquefaciens*.** (A) Schematic illustration of the confrontation zone between *C. graminicola* and *B. amyloliquefaciens*, with colored rectangles indicating sampling sites at increasing distances of the fungal hyphae from the bacterial colony. (B) Percentage of the area covered by hyphal protrusions at different distances from the colony edge of *C. graminicola*. Error bars are +SDs.


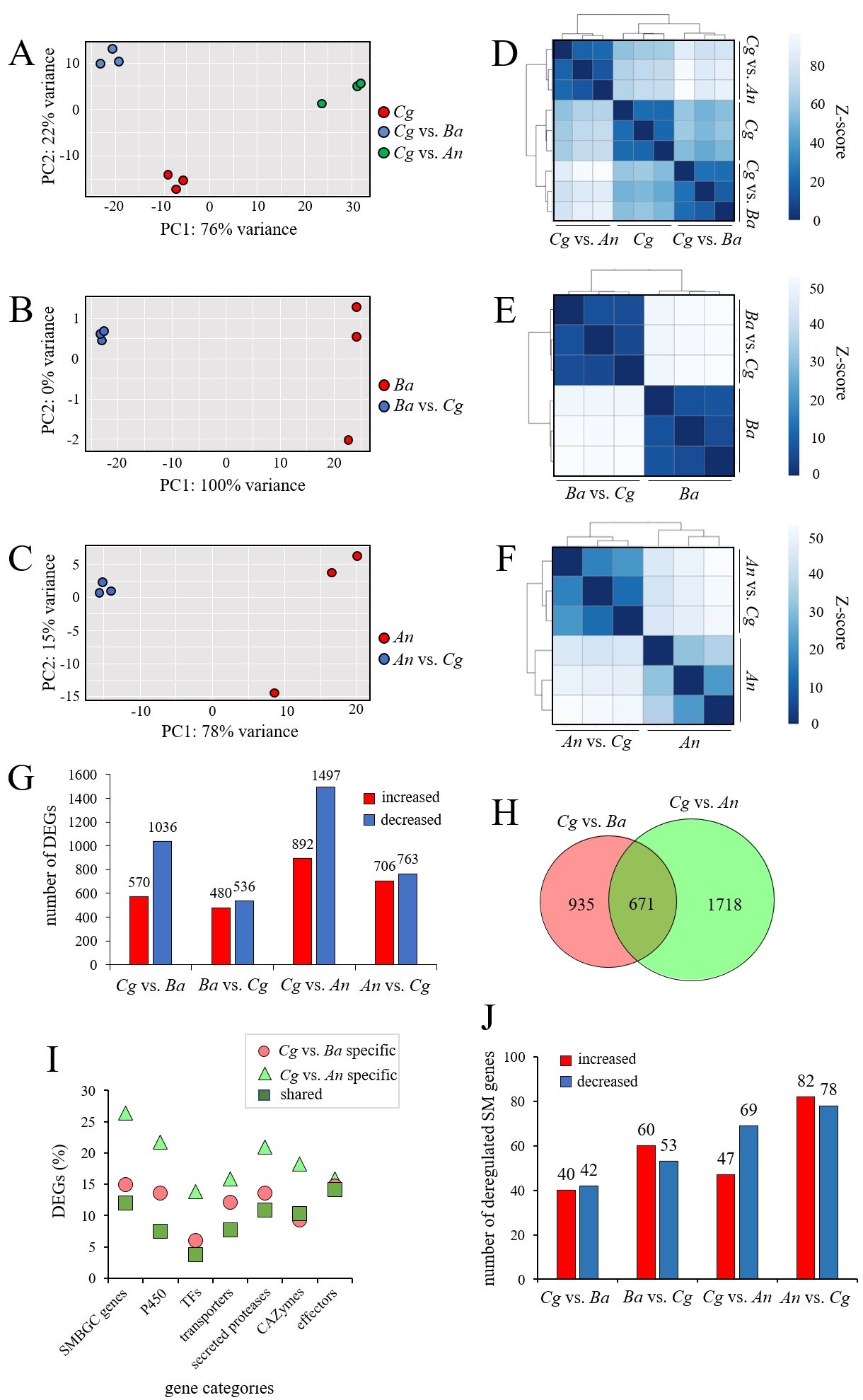


**Figure S4. Transcriptome analysis of differentially expressed genes (DEGs) under confrontations.** (A) Principal component analyses (PCA) show clear cluster separation of *C. graminicola* solo cultures and hyphae confronting *B. amyloliquefaciens* (*Cg* vs. *Ba*) or *A. nidulans* (*Cg* vs. *An*). (B) PCA plot showing distinctness of *B. amyloliquefaciens* monocultures and cultures confronting *C. graminicola* (*Ba* vs. *Cg*). (C) PCA plot showing distinctness of monocultures of *A. nidulans* and mycelia confronting *C. graminicola* (*An* vs. *Cg*). Sample groups are indicated by different color codes. Each replicate is plotted as an individual data point. (D) Heatmap of the sample-to-sample distance matrix obtained from monoculture of *C. graminicola* and confrontations with *B. amyloliquefaciens* and *A. nidulans.* (E) Heatmap of the sample-to-sample distance matrix of *B. amyloliquefaciens* monocultures and cultures confronting *C. graminicola.* (F) Heatmap of the sample-to-sample distance matrix of *A. nidulans* monocultures and cultures confronting *C. graminicola.* The color codes in sub-figures (D – F) indicate the distance between the samples, as based on the Z-score. Dark blue denotes shorter distance i.e., replicates are grouped closer in distance. (G) DEGs identified in different confrontations and confrontation partners. Red and blue bars indicate increased (FC > 2) and decreased (FC < 0.5) transcript abundances with adjusted p-value < 0.05. (H) Venn diagram representing the distribution of the DEGs of *C. graminicola* confronting *B. amyloliquefaciens* (*Cg* vs. *Ba*) or *A. nidulans* (*Cg* vs. *An*). The number in the overlap denotes the mutual DEGs between distinct confrontations. (I) Scatter plot showing confrontation-specific and shared DEGs of *C. graminicola*. Genes are grouped according to functional categories. (J) Bar graph showing the number of SM genes deregulated in different confrontation partners in distinct confrontations. Red and blue bars indicate increased and decreased transcript abundances of differentially expressed SM genes.

**
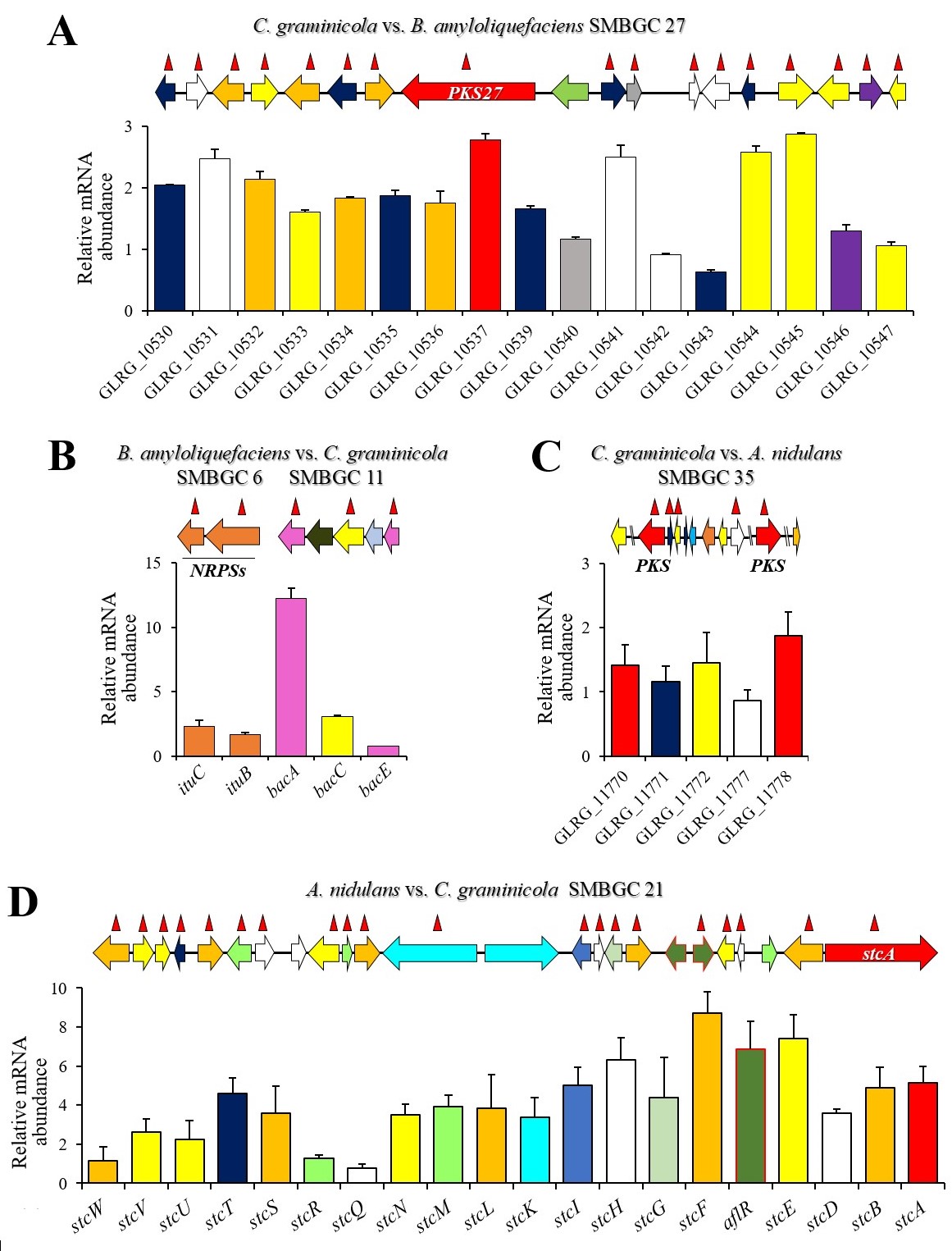
**

**Figure S5. Validation of RNA-Seq data by reverse transcription- quantitative polymerase chain reaction (RT-qPCR) as an independent method.** (A) Seventeen genes of SMBGC 27 of *C. graminicola* showing increased transcript abundances in confrontation with *B. amyloliquefaciens* were validated by RT-qPCR. (B) As for the RNA-Seq analyses, two iturin and three bacilysin genes of SMBGCs 6 and 11 in *B. amyloliquefaciens* showed increased transcript abundances in confrontation with *C. graminicola*. (C) Confirmation of increased transcript abundances of the five genes of SMBGC35 of *C. graminicola* confronting *A. nidulans*. (D) Twenty out of 24 genes of the sterigmatocystin genes of SMBGC 21 of *A. nidulans* exhibited increased transcript abundances also when analyzed by RT-qPCR.

The physical maps of the corresponding SMBGCs and genes with increased transcript abundances, as analyzed by RNA-Seq studies, are given above the respective bar plot. The constitutively expressed actin biosynthesis genes of *C. graminicola* and *A. nidulans*, as well as the *gyrA* gene of *B. amyloliquefaciens* served as standards. Data shown are the means of three independent biological replicates and three technical replicates. Error bars are +SDs.

**
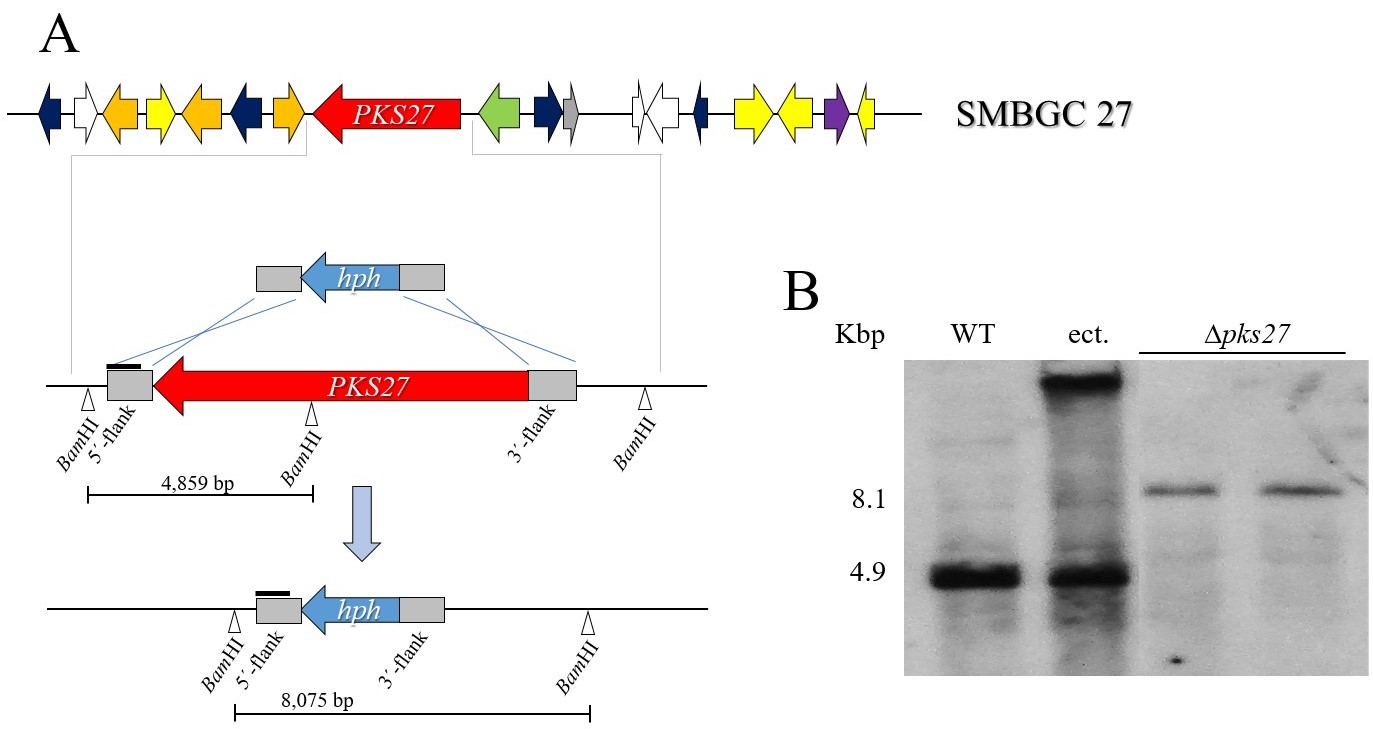
**

**Figure S6. Targeted deletion of *PKS27* of *C. graminicola*.** (A) *PKS27* of SMBGC 27, encoding a polyketide synthase, was replaced by a construct consisting of the hygromycin phosphotransferase gene *hph* of *E. coli* and the 5’- and 3’-flanks of *PKS27* of *C. graminicola*. *Bam*HI sites and size of DNA fragments are indicated. The position of the probe is indicated as a black line over the 5’-flank. (B) Genomic Southern blot of *Bam*HI-digested genomic DNA of the wildtype (WT), an ectopic (ect.) and two Δ*pks27* strains. The blot was hybridized with the 5’-flank-specific probe.

**
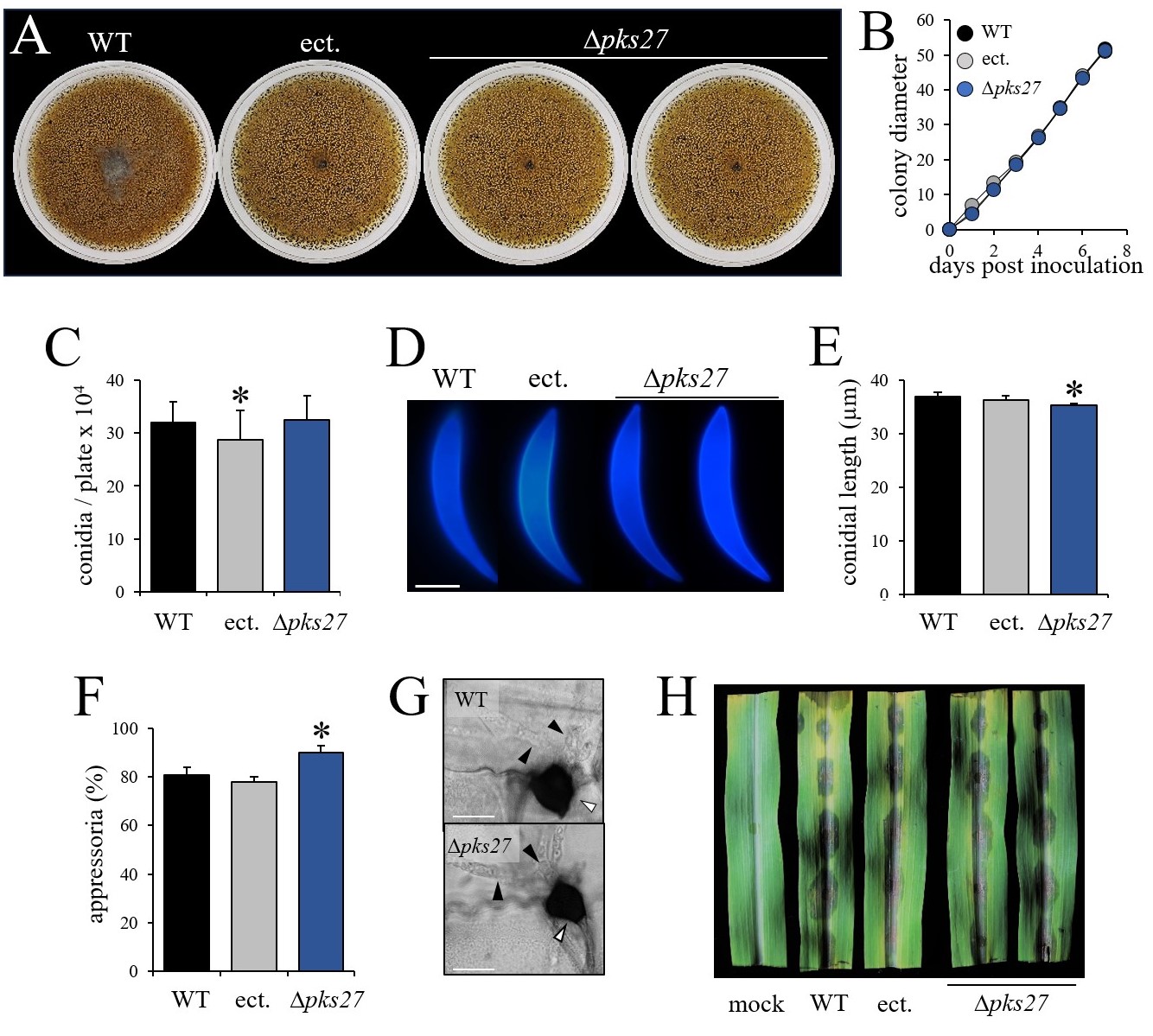
**

**Figure S7. *PKS27* of *C. graminicola* is not required for vegetative growth, conidiation or virulence.** The colony phenotype (A) as well of radial growth rates (B) of WT, ectopic (ect.) and ∆*pks27* strains grown on PDA are not discernible. Photographs in (A) were taken at 14 dpi. Growth rates (B) were measured daily. (C) WT, ectopic (ect.) and ∆*pks27* strains had formed comparable numbers of conidia onto PDA at 14 dpi. (D) The shape of the conidia was not altered by deletion of *PKS27*, but (E) the length of conidia of ∆*pks27* strains was marginally reduced. (F) The percentage of appressoria differentiated from falcate conidia on maize (cv. Mikado) leaf surfaces was slightly but statistically significantly (P ≤ 0.05) higher in ∆*pks27* strains, but (G) appressoria (white arrowheads) of both WT and ∆*pks27* strains invaded the host epidermal cells and formed normal biotrophic hyphae (black arrowheads). (H) WT, ectopic (ect.) and ∆*pks27* strains elicited comparable disease symptoms.

**
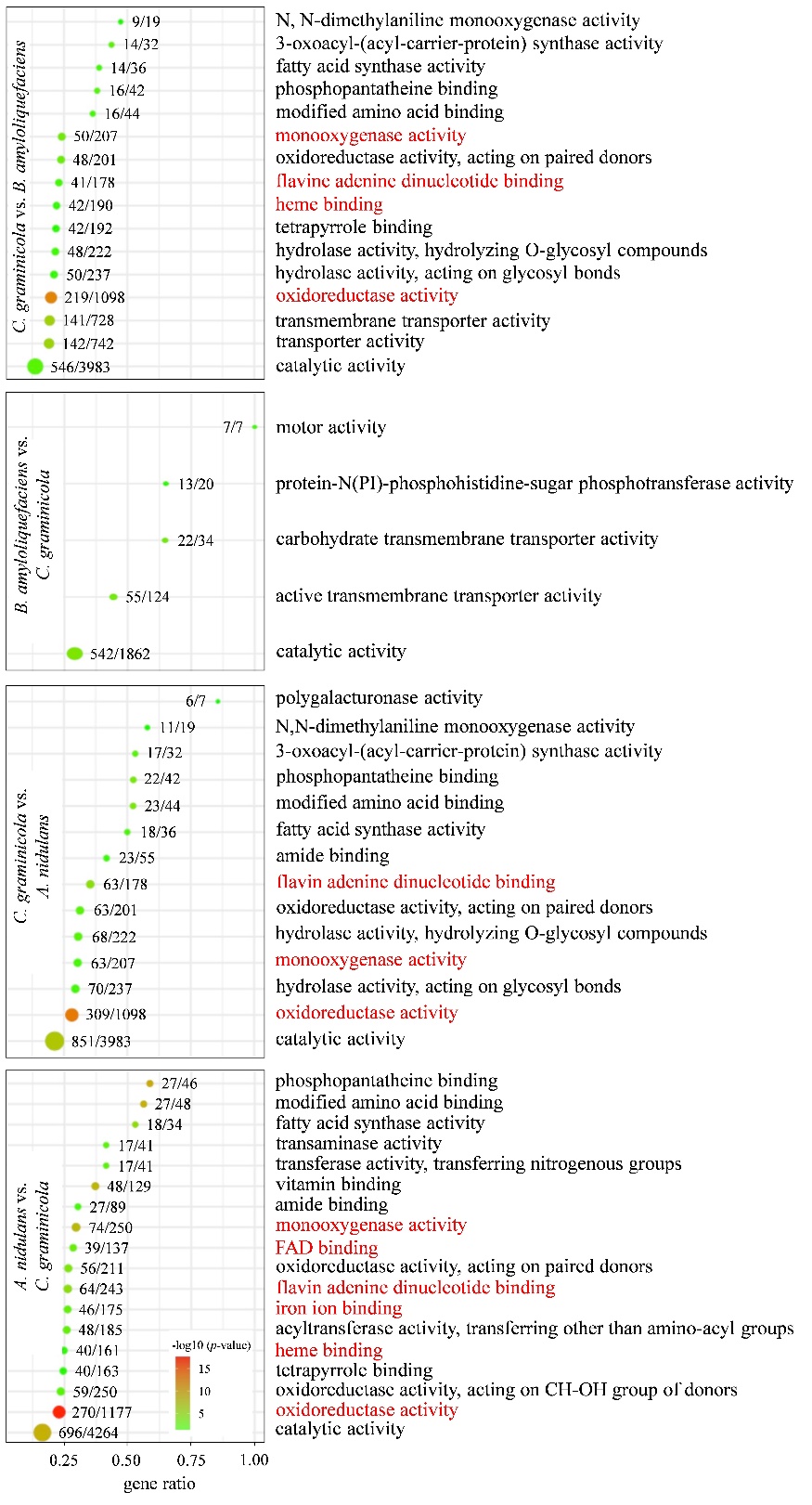
**

**Figure S8.** Gene Ontology (GO) enrichment analysis of differentially expressed genes (DEGs) under microbial confrontations. Bubble plots display enriched GO molecular function terms among DEGs identified during pairwise microbial confrontations. The X-axis represents the gene ratio, defined as the number of DEGs associated with a GO term divided by the total number of genes annotated to that term. Bubble sizes indicate the number of DEGs mapped to each GO term. Bubble color corresponds to statistical significance, represented as the negative log10 of the adjusted p-value. Only GO terms with adjusted p-values less than 0.05 are included.

**
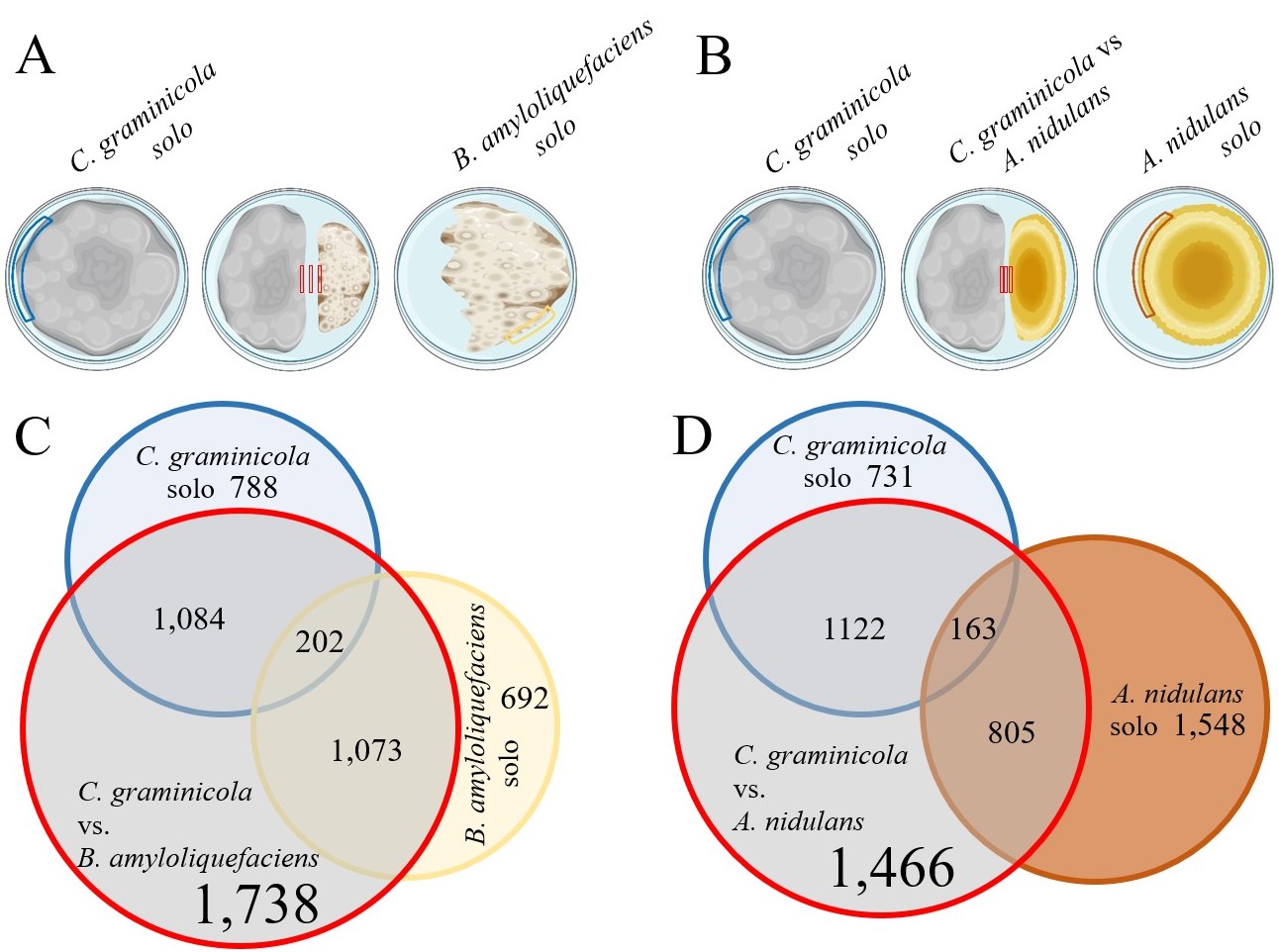
**

**Figure S9. Sample collection for metabolome analyses and Venn diagram showing numbers of chemistries newly synthesized in distinct confrontations.** (A and B) Samples collected from 5 mm of the confronting culture margins and from the area between cultures lacking fungal or bacterial cells (red rectangles). Samples from margins of monocultures (blue, yellow and brown marked areas) served as controls. Blue and yellow areas denote the sample isolation spots in solo-cultures, and red area in co-culture denotes the sample isolation from confrontation partners and inhibition zone. (C and D) Venn diagram showing 1,738 molecules specifically synthesized in the confrontation between *C. graminicola* and *B. amyloliquefaciens*, and 1,466 in the *C. graminicola* vs. *A. nidulans* confrontation.


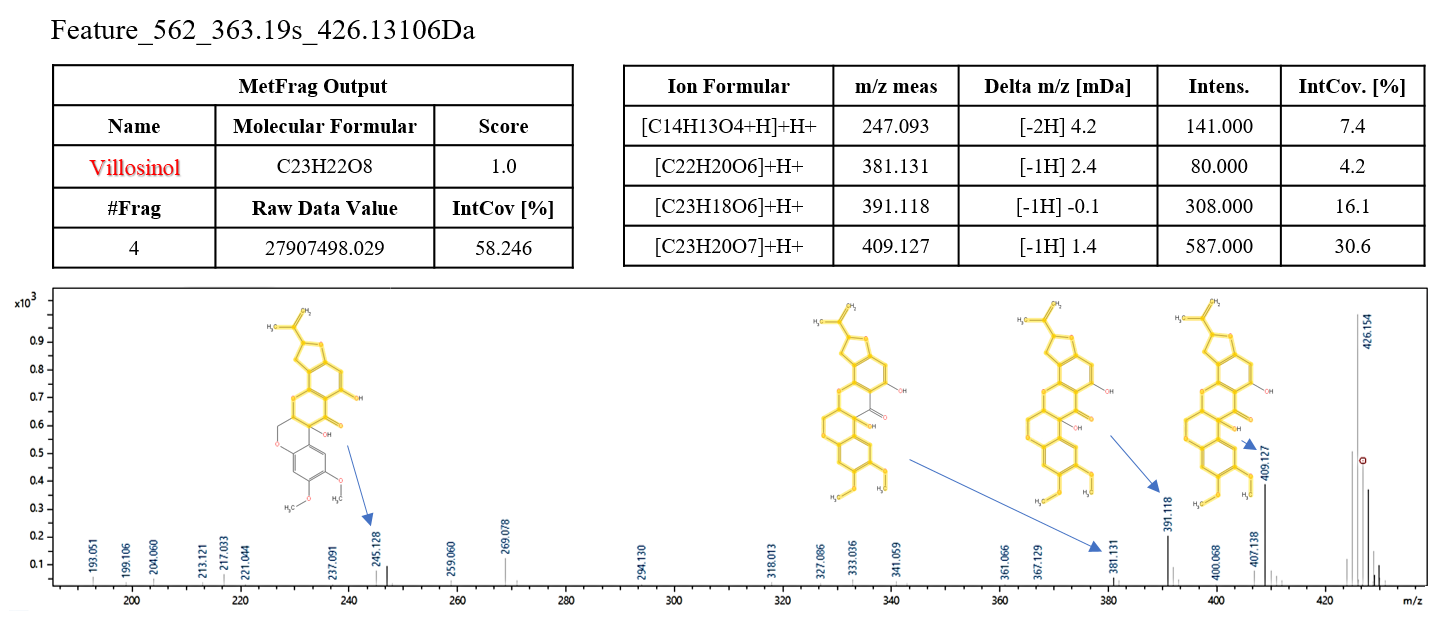


**Figure S10. Annotation of compound feature 562 from the C. graminicola – A. nidulans confrontation using MetFrag software.** The compound is produced in hyphae of *A. nidulans* (see Figure 5D, isoflavonoids, profile 1) and was annotated as villosinol. The MS/MS spectra, with a retention time (RT) of 363.19 seconds and an m/z value of 427.14, revealed four characteristic fragments (m/z 247.093; 381.131; 391.118; and 409.127). These fragments correspond to the stepwise loss of chemical groups from the villosinol molecule. The structures of these fragments are illustrated alongside the MS/MS spectrum.


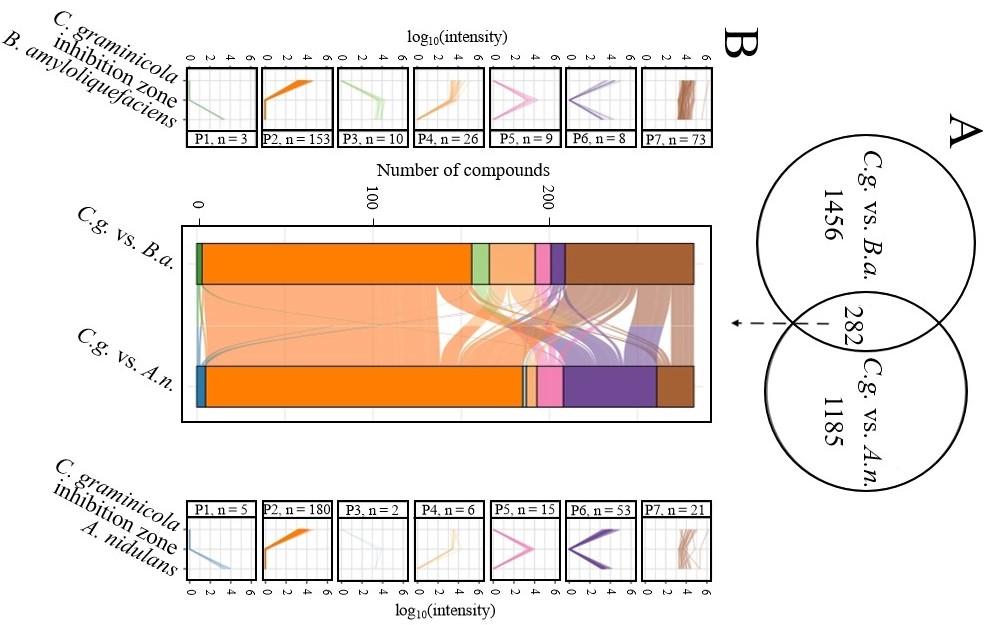


**Figure S11. Confrontation- and profile-specificity of features synthesized in the confrontations of *C. graminicola* with *B. amyloliquefaciens* or *A. nidulans*.** (A) Venn diagram indicating that of the 1,738 and 1,466 features synthesized in the fungus – bacterium and in the fungus – fungus confrontation, respectively. Only 282 were common to both interactions. (B) Alluvial plot showing comparison of the 282 features shared between both confrontations. Profiles P1 – P7 are as in Figures 5 and 6. Stacked bar plots for each of the confrontations show the number of features per subset, with colors corresponding to profile plots. Lines connect the same individual features.
